# Cowpea and abiotic stresses: identification of reference genes for transcriptional profiling by qPCR

**DOI:** 10.1186/s13007-018-0354-z

**Published:** 2018-10-12

**Authors:** Lidiane Lindinalva Barbosa Amorim, José Ribamar Costa Ferreira-Neto, João Pacífico Bezerra-Neto, Valesca Pandolfi, Flávia Tadeu de Araújo, Mitalle Karen da Silva Matos, Mauro Guida Santos, Ederson Akio Kido, Ana Maria Benko-Iseppon

**Affiliations:** 1 Instituto Federal de Educação, Ciência e Tecnologia do Piauí, Oeiras, Piauí Brazil; 2 Genetics Department, Universidade Federal de Pernambuco, Recife, Pernambuco Brazil; 3 Botany Department, Universidade Federal de Pernambuco, Recife, Pernambuco Brazil

**Keywords:** Housekeeping genes, Root tissue, Salt stress, Drought, Phenylpropanoids, Legume

## Abstract

**Background:**

Due to cowpea ability to fix nitrogen in poor soils and relative tolerance to drought and salt stresses, efforts have been directed to identifying genes and pathways that confer stress tolerance in this species. Real-time quantitative PCR (qPCR) has been widely used as the most reliable method to measure gene expression, due to its high accuracy and specificity. In the present study, nine candidate reference genes were rigorously tested for their application in normalization of qPCR data onto roots of four distinct cowpea accessions under two abiotic stresses: root dehydration and salt (NaCl, 100 mM). In addition, the regulation of four target transcripts, under the same referred conditions was also scrutinized.

**Results:**

geNorm, NormFinder, BestKeeper, and ΔCt method results indicated a set of three statistically validated RGs for each stress condition: (I) root dehydration (actin, ubiquitin-conjugating enzyme E2 variant 1D, and a *Phaseolus vulgaris* unknown gene—*UNK*), and (II) salt (ubiquitin-conjugating enzyme E2 variant 1D, F-box protein, and *UNK*). The expression profile of the target transcripts suggests that flavonoids are important players in the cowpea response to the abiotic stresses analyzed, since chalcone isomerase and chalcone synthase were up-regulated in the tolerant and sensitive accessions. A lipid transfer protein also participates in the cowpea tolerance mechanisms to root dehydration and salt stress. The referred transcript was up-regulated in the two tolerant accessions and presented no differential expression in the sensitive counterparts. Chitinase B, in turn, generally related to plant defense, was an important target transcript under salt stress, being up-regulated at the tolerant, and down-regulated in the sensitive accession.

**Conclusions:**

Reference genes suitable for qPCR analyses in cowpea under root dehydration and salt stress were identified. This action will lead to a more accurate and reliable analysis of gene expression on this species. Additionally, the results obtained in this study may guide future research on gene expression in cowpea under other abiotic stress types that impose osmotic imbalance. The target genes analyzed, in turn, deserve functional evaluation due to their transcriptional regulation under stresses and biotechnological potential.

**Electronic supplementary material:**

The online version of this article (10.1186/s13007-018-0354-z) contains supplementary material, which is available to authorized users.

## Background

Cowpea [*Vigna unguiculata* (L.) Walp] is one of the most important legumes cultivated by subsistence farmers, for both human and livestock consumption, mainly in the semi-arid regions of Africa [1] and Brazil [2]. In Africa, it is used for the livelihoods of millions of people in the semi-arid regions of West and Central regions [3] and is considered the most important grain legume crop in the sub-Saharan region. Regarding its potential for grain supply in dry areas (due to its wide genetic variability and good nitrogen fixation capacity), this crop minimizes the almost exclusive dependence on common bean, conferring to cowpea a strategic value in Brazil [2].

Given the cowpea great economic and socio-cultural importance, in addition to its rusticity and phenotypic plasticity on adverse soil and climatic conditions (with peculiar features of tolerance/resistance to stresses), the *Cowpea Functional Genome Consortium* (CpFGC) was created [4]. Using different methodological approaches (i.e. ESTs, HT-SuperSAGE and RNA-Seq) this effort resulted in the generation of millions of transcripts obtained from cowpea plants under different abiotic [root dehydration and salt (NaCl, 100 mM)] and biotic (infection by Cowpea Aphid-borne Mosaic Virus—CABMV and Cowpea Severe Mosaic Virus—CPSMV) stresses [4]. These data provide a good start for identifying putative genes and gene families associated with resistance/tolerance to such challenging conditions [4].

Real-time quantitative PCR (qPCR) is the most commonly used method of validation of transcriptomic studies, due to its high sensitivity and specificity [5]. Despite the advantages, its reliability depends on various factors, such as the integrity and purity of RNA, the efficacy of various reagents and enzymes used in RNA extraction, sample quantification, and reverse transcription, among others [5]. Such variables can cause quantitative and qualitative differences between the analyzed samples. Thus, the careful planning of protocols is mandatory when implementing qPCR, and the use of reference genes (RGs) as normalizers is an essential prerequisite. RGs should ideally be constitutively expressed in the studied tissue or cell type and should not be affected by the treatments performed. Additionally, uniform transcript abundance across the different groups being analyzed (e.g., across treatments) is necessary, serving as a “calibrator” to compare the different samples of the same quantitative level. This way, the use of suitable RGs ensure that the observed variation in the relative quantification of the target transcripts is due to changes in the gene expression, avoiding false positives or negatives in the transcriptome analysis.

Housekeeping genes are required for basal procedures and cellular survival, being often stably expressed and therefore used as normalizers without validating their suitability [6]. Nevertheless, increasing evidence showing that the transcription level of commonly used RGs (such as 18S ribosomal RNA, 25S ribosomal RNA, glyceraldehyde-3-phosphate dehydrogenase, elongation factor 1-alpha, among others [7, 8]) can vary considerably depending on the state of development and physiological conditions, especially under stress. Therefore, various statistical methods have been developed to validate the expression stability of candidate RGs, and to allow the selection of the most suitable candidates for particular condition/tissue/species. NormFinder [9], geNorm [10] and BestKeeper [11] algorithms, besides the ΔCt method [12], are the most widely used approaches.

Unfortunately, only a limited number of studies addressing gene expression and qPCR have been reported for cowpea so far. Coetzer et al. [13] used the glyceraldehyde-3-phosphate dehydrogenase C-subunit gene as normalizer in the evaluation of target transcripts in leave tissues of cowpea plants under drought. Mellor et al. [14] used the actin gene as normalizer to evaluate *RSG3*-*301* (acronym of “resistance to *S. gesnerioides* race 3”) transcript levels involved in the resistance response of transgenic cowpea roots (Blackeye cultivar) to the attack by the root parasitic weed, *Striga gesnerioides.* Huang et al. [15] also used the actin gene as an endogenous control to analyze target genes expression of cowpea (cultivar B301) during compatible and incompatible interactions with different races (SG3 and SG4z) of *Striga gesnerioides*. In turn, Shui et al. [16], used the small nuclear RNA U6 gene as an endogenous control to analyze target microRNAs in the leaves and roots of cowpea plants under drought treatment. The studies above have in common the fact that the respective RGs employed were not previously subjected to careful statistical analysis to determine their stability, following statistical tests, in the condition/tissue/plant analyzed. In addition, only one reference gene was used in each proposed assay, which reduces the statistical robustness of the results. According to MIQE (Minimum Information for publication of Quantitative real-time PCR Experiments) guidelines [5], which presents a series of indications on good practices in experiments involving qPCR, normalization should be carried out against multiple RGs, chosen from a variety of candidate RGs tested from independent pathways with the application of at least one algorithm [5, 10]. However, few works have focused on the selection of RGs in cowpea based on different statistical software. The first study was conducted by Da Silva et al. [17], who evaluated the expression stability of eight candidate genes in cowpea under drought stress during biological nitrogen fixation, using geNorm and NormFinder algorithms. The genes of the regulatory subunit of phosphatase 2A protein (*VuPp2A*) and polyubiquitin 28 (*VuUbq28*) were the best normalizers suggested by both algorithms, for global analysis (nodules and leaves tissues).

The present study was undertaken to select and validate suitable RGs tested for effective normalization of cowpea qPCR data. Root tissues of four contrasting cowpea accessions (tolerant and sensitive), under two different abiotic stresses [root dehydration or salt (NaCl, 100 mM)] were analyzed through three different algorithms (NormFinder [9], geNorm [10], BestKeeper [11]), and also the ΔCt method [12], to find the most suitable RGs for each experimental condition. In addition, the transcriptional regulation of four cowpea target transcripts [chalcone isomerase (*VuCHI*), chalcone synthase (*VuCHS*), lipid transfer protein (*VuLTP*), and chitinase B (*VuCHiB*)] was scrutinized. The gene expression data obtained from a Next Generation Sequencing approach (HT-SuperSAGE) has been used to evaluate the participation of these targets in response to the studied stresses. The proposed RGs will serve to validate RNA-Seq and HT-SuperSAGE data generated by the CpFGC, as well as will benefit future studies on gene expression in cowpea and related species.

## Methods

### Plant material and treatments (root dehydration and salt stresses)

Two independent experimental trials were performed in the present study: one with root dehydration **(**Additional file 1: Fig. S1A**)** and the other with salt stress (Additional file 1: Fig. S1B). For root dehydration assay (Additional file 1: Fig. S1A), cowpea cultivars ‘Pingo de Ouro’ (PO; drought-tolerant) and ‘Santo Inácio’ (SI; drought-sensitive) were grown in a greenhouse at Embrapa-Soybean station (Londrina, Brazil), under hydroponic conditions (30 L plastic containers, pH 6.6, and balanced nutrient solution, as reported by Kulcheski et al. [18]). Briefly, cowpea seedlings, with the first trifoliate leaf fully developed, were submitted to root dehydration (in the dark) for 0 minutes (negative control), 25 (T1), 50 (T2), 75 (T3), 100 (T4), 125 (T5), and 150 (T6) minutes (Additional file 1: Fig. S1A) after removal of the nutrient solution from the tray. At the end of each treatment, the roots were frozen in liquid nitrogen and stored at − 80 °C until total RNA extraction.

For salt stress (Additional file 1: Fig. S1B), seeds of two contrasting cultivars, named ‘Pitiúba’ (PI; salt-tolerant; [19, 20]) and ‘BR14-Mulato’ (BR; salt-sensitive; [21]) were grown in pots with washed sand watered with 200 ml of 1/2 strength Hoagland’s Solution. Seedlings (with first trifoliate leaf fully developed) were submitted to different periods of exposition to salt (NaCl added to the Hoagland’s Solution to a final concentration of 100 mM). Roots were collected at 0 minutes (negative control; only with Hoagland Solution and water), 30 (T1), 60 (T2) and 90 (T3) minutes after irrigation with saline (NaCl, 100 mM) Hoagland’s Solution (Additional file 1: Fig. S1B).

The treatment times mentioned in the trials were distinct because the events were independent, and these treatments took into account physiological analyzes (manuscript in preparation) indicating that the plants begin to undergo stress effects. The experimental designs were factorials (cultivars × extent of the stress) with three biological replicates (five plantlets composed each replicate).

### Total RNA isolation and cDNA synthesis

Total RNA of cowpea root tissues (in all cultivars and treatments) was isolated using ‘SV Total RNA Isolation System Kit’ (Promega, Madison, WI) following the manufacturer’s instructions. The genomic DNA (gDNA) was eliminated by RNase-free DNase I digestion during the isolation procedure. The quantity and quality of the isolated RNA were evaluated, respectively, using a NanoDrop ND-1000 UV–Vis Spectrophotometer (Thermo Fisher Scientific) and by electrophoresis agarose gels 1% (w/v), stained with Blue Green (LGC, São Paulo, Brazil). For each sample, the total RNA (1 µg) was reverse-transcribed into cDNA, using the ‘Improm-II™ Reverse Transcriptional System’ (Promega) with oligo (dT) primers following the manufacturer’s recommendations. The quality of each cDNA was assessed by using standard PCR reaction with an actin primer pair [F: GGAACATCCCGTTCTCTTGA and R: CTCTCAGGAGGAGCAACCAC, amplicon of 708 bp; template Contig16004 (CpFGC database; Additional file 2: S1 Appendix)] that spanned intronic regions.

### HT-SuperSAGE libraries, statistical analysis, and unitag-gene annotation

The HT-SuperSAGE libraries were synthesized for root dehydration and salt stresses. For root dehydration assay, two HT-SuperSAGE libraries were generated for each cultivar: POT1-6 (drought-tolerant cultivar under stress—a bulk with similar amounts of RNAs poly A^+^ from samples covering the six stress times; Additional file 1: Fig. S1A), and POT0 (drought-tolerant cultivar, negative control; Additional file 1: Fig. S1A); SIT1-6 (drought-sensitive cultivar under stress—bulk of six stress times; Additional file 1: Fig. S1A), and SIT0 (drought-sensitive cultivar, negative control; Additional file 1: Fig. S1A). The salt stress included the following libraries: PTS3T (salt-tolerant cultivar Pitiúba under stress—bulk of three stress times; Additional file 1: Fig. S1B), and PTT0 (salt-tolerant cultivar, negative control; Additional file 1: Fig. S1B); BRS3T (salt-sensitive accession BR14-Mulato under stress—bulk of three stress times), and BRT0 (salt-sensitive cultivar, negative control; Additional file 1: Fig. S1B). All HT-SuperSAGE libraries were generated at GenXPro GmbH (Frankfurt, Germany) as described by Matsumura et al. [22] and submitted to Illumina sequencing technology.

HT-SuperSAGE tags (26-bp) were analyzed to find unique and differentially expressed unitags (*p *< 0.05) based on Poisson statistics developed by Audic and Claverie [23], as implemented in DiscoverySpace (v.4.01) software [24]. The singlets (tags sequenced only once) were excluded from the evaluation. Unitags were annotated by BLASTn against nucleotide sequences from *Vigna unguiculata* available at Phytozome Database (https://phytozome.jgi.doe.gov/pz/portal.html#). The BLASTn alignments (unitag-EST) with e-value ≤ 0.001 and scores higher than 50 (i.e., with a maximum of one mismatch) were identified between the plus/plus matches. Unitags with mismatch regarding the four first bases “CATG” were not accepted to guarantee the integrity of the unitags.

### Selection of the candidate RGs, target transcripts and primers design

The workflow of qPCR assay and stability analysis of the RGs is depicted in Additional file 3: Fig. S2. For the selection of the candidate RGs, a literature search in PubMed Database (https://www.ncbi.nlm.nih.gov/pubmed/) was carried out using the terms ‘*Phaseolus vulgaris* AND qPCR’; ‘*Vigna unguiculata* AND qPCR’. In addition, a data mining for candidate RGs also was performed in the CpFGC database.

Nine candidates were selected for the present study (Table 1) including:

**Table 1:** Candidate reference genes, target transcripts and respective primers pairs used in the present work

Gene	Anchor specie^*^	Cellular function	Primer sequences	Amplicon size (bp)	References
*VuACT*	*Vigna unguiculata* (Contig16004)	Diverse functions, ranging from cell motility to maintenance of cell shape and polarity	F: TCAGGTGTCCAGAGGTGTTGTA R: ATGGTTGTGCCTCCTGAAAGTA	151	CpFGC Database
*VuUBQ10*	*Vigna unguiculata* (Contig282)	Protein ubiquitination pathway	F: GTCTAAGGGGAGGAATGCAGAT R: CAAAGATCAACCTCTGCTGGTC	150	CpFGC Database
*β*-*TUB*	*Phaseolus vulgaris* (XM_007147394.1)	Internal cell architecture maintenance drives cytoplasmic streaming and others	F: CCGTTGTGGAGCCTTACAAT R: GCTTGAGGGTCCTGAAACAA	117	[25]
*EF1*-*α*	*Phaseolus vulgaris* (XM_007151727.1)	Enzymatic release of aminoacyl tRNAs to the ribosome	F: GGTCATTGGTCATGTCGACTCTG R: GCACCCAGGCATACTTGAATGACC	146	[26]
*FBOX*	*Phaseolus vulgaris* (XM_007131876.1)	Mediation of protein–protein interaction	F: CACCAGGATGCAAAAGTGG R: ATCCGCTTGTCCCTTGAAC	163	[27]
*UE21D*	*Phaseolus vulgaris* (XM_007145751.1)	Protein ubiquitination pathway; DNA repair pathway	F: AGAAAAGCCCCCAAGTGTTC R: CTGCCATCTCCTTCTTCAGC	161	[27]
*UNK*	*Phaseolus vulgaris* (XM_007131494.1)	Unknown function, putatively a membrane-associated protein	F: ATTCCCATCATGCAGCAAAG R: AGATCCCTCCAGGTCAATCC	192	[27]
*ZMP*	*Phaseolus vulgaris* (XM_007162147.1)	Metalloproteinase (i.e., protease enzyme whose catalytic mechanism involves a metal)	F: GCAACCAACCTTTCATCAGC R: AGAAATGCCTCAACCCTTTG	156	[27]
*GAPC*	*Glycine max* (XM_003526927.3)	Catalyzes an essential energy-yielding step in carbohydrate metabolism	F: ATCAGCCAAGGACTGGAGAG R: ACGGAATGCCATACCAGTCA	130	[13]
*VuCHiB*	*Vigna unguiculata* (Contig5335)	Hydrolytic enzyme that breaks down glycosidic bonds in chitin. It plays an important role not only in plant defense but also in various abiotic stresses	F: CCATCTGGTTCTGGATGACC R: CCGTTGATGATGTTCGTCAC	130	CpFGC Database
*VuLTP*	*Vigna unguiculata* (Contig14261)	Play important roles in biotic and abiotic stresses responses	F: TGTGATGATGGAAGCGAATG R: TGAGCAGCAATCAGAGGTTG	124	CpFGC Database
*VuCHI*	*Vigna unguiculata* (Contig12804)	Catalyzes the conversion of naringenin chalcone to naringenin and is strictly required for flavonoid production	F: CACATACCATTTCCCAGCAG R: TGGAAGACACTGCCCTTGAG	149	CpFGC Database
*VuCHS*	*Vigna unguiculata* (Contig7106)	Catalyzes the first committed step in the flavonoid biosynthetic pathway	F: GACTGCACAGACCATTGCAC R: GGATCGAAGGCTTCAGAAAG	144	CpFGC Database

**Candidate reference genes** (*VuACT*: actin; *VuUBQ10*: polyubiquitin 10; *β*-*TUB*: beta-tubulin; *EF1*-*α*: elongation factor 1-alfa; *FBOX:* F-box protein; *UE21D*: ubiquitin-conjugating enzyme E2 variant 1D; *UNK*: *Phaseolus vulgaris* unknown gene; *ZMP*: zinc metalloproteinase; *GAPC*: glyceraldehyde-3-phosphate dehydrogenase C-subunit). **Target transcripts** (*VuCHiB*: chitinase B; *VuLTP*: lipid transfer protein; *VuCHI*: chalcone isomerase; *VuCHS*: chalcone synthase). *Vu* (*Vigna unguiculata*). *Based on RefSeq-NCBI and Cowpea Functional Genome Consortium (CpFGC) databases

- six (*β*-*TUB*: beta-tubulin; *EF1*-*α*: elongation factor 1-alfa; *FBOX*: F-box protein; *UE21D*: ubiquitin-conjugating enzyme E2 variant 1D; *UNK*: *Phaseolus vulgaris* unknown gene; and *ZMP*: zinc metalloproteinase) anchored in *Phaseolus vulgaris* genes;
- one (*GAPC*: glyceraldehyde-3-phosphate dehydrogenase C-subunit) anchored in soybean (*Glycine max*) gene;
- and two candidate RGs (*VuACT*: actin and *VuUBQ10*: polyubiquitin 10) anchored in *Vigna unguiculata* genes, previously designed by our group (CpFGC, Cowpea Functional Genome Consortium).

The candidate RGs obtained from in *V. unguiculata* genes were identified by BLASTn search (cutoff < e^−10^) in CpFGC database, using predicted *Vigna radiata* (actin) and *Arabidopsis* (ubiquitin10) genes as queries (Additional file 2: S1 Appendix). All candidates were also selected based on their involvement in diverse plant cellular processes reducing, thus, the probability of co-regulation.

The primer pairs were designed using the online Primer3 software (http://bioinfo.ut.ee/primer3-0.4.0/) with the following parameters: annealing temperature of 57–63 °C (optimal 60 °C), primer length of 18–22 bp (optimal 20 bp), GC contents of 45–55% (optimal 50%) and amplicon length of 100–200 bp (Table 1).

The target transcripts (*VuCHiB*, *VuLTP*, *VuCHI*, *VuCHS*; Table 1), whose expression was analyzed in the present work, were chosen because of their presence in both analyzed assays (root dehydration and salt stress HT-SuperSAGE libraries) and up-regulation (FC > 5; p < 0.05) in the ‘Pingo de Ouro’ accession, considered tolerant to the drought stress. Fold change (FC) is a measure describing how the unitag expression modulated after the stress. The FC values were based on the ratio (R) of the normalized unitag frequencies considering two libraries (treatment and control). In the case of R < 1, the FC = − 1/R, being the negative FC values indicator of repressed unitags; in the case of ‘zero’ frequency in a library, this value was replaced by ‘one.’

### qPCR setup, amplification efficiency, and relative expression analysis

Although the root dehydration assay covered six exposition stress times, three of them (25, 75 and 150 min.) was chosen for the RTqPCR data validation, representing the initial, intermediate, and late stress exposition times, respectively. For salt stress, the studied points were 30, 60 and 90 min. Three biological and three technical replicates per sample were used to ensure statistical reliability. The same number was used for the not stressed controls maintained under the same condition. The qPCR reactions were performed in 96-well plates and performed on the LineGene 9660 (Bioer), using SYBR Green detection. Reactions were prepared in a total volume of 10 μL containing: 1 μL of 10 fold diluted template, 5 μL ‘HotStart-IT SYBR Green qPCR Master Mix 2x’ (USB), 0.05 μL of ROX, 1 μL of each primer (500 nM) and nuclease-free water to a final volume of 10 μL. The PCR program was adjusted to 95 °C for 2 min, followed by 40 cycles of 95 °C for 15 s, 58 °C for 30 s, and 72 °C for 15 s. After amplification, dissociation curves were produced (60–95 °C at a heating rate of 0.1 °C/sec and acquiring fluorescence data every 0.3 °C) to confirm the specificity of the PCR products.

The amplification efficiency (E = 10^(−1/slope of the standard curve)^; Additional file 3: Fig. S2) for all primer pairs was determined from a 5-point standard curve generated by serial dilutions of cDNA (10-fold each) in technical triplicates. Slopes in the range of -3.58 to -3.10 were considered acceptable for the qPCR assay [28]. These slope values correlated to amplification efficiencies between 90% (E = 1.9) and 110% (E = 2.1; Additional file 3: Fig. S2).

The Rest2009 software package (standard mode) was used for relative expression analysis of target transcripts. REST bases its performance on pairwise comparisons (of target transcripts and reference genes) using randomization and bootstrapping techniques—Pair-wise Fixed Reallocation Randomization Test© [29, 30]. Hypothesis testing (p < 0.05) was used to determine whether the differences in target transcripts expression between the control and treatment conditions were significant.

### Statistical analyses of candidate RGs expression stability

The expression stability of each candidate RG was evaluated by four different strategies **(**Additional file 3: Fig. S2**).**

GeNorm algorithm [10] calculates an expression stability value (M) for each candidate RG. Then, the algorithm determines the pairwise variation (V) of each candidate RG with all of the others studied. At the end of the analysis, by stepwise exclusion of the gene with the highest M-value (less stable), this tool allows for the ranking of the tested RGs according to their expression stability. The optimal number of RGs required for normalization was determined by pairwise variation V_n_/V_n + 1_. According to Vandesompele et al. [10], a cut-off value of V_n/n+1_ < 0.15 dispense the inclusion of additional RG. Despite the possibility to achieve this prerequisite with the use of only two stable RGs, it is recommended the use of at least three reference genes (NF, *n* = 3) for calculation of a qPCR normalization factor [10].

The NormFinder [9] algorithm provides a stability value (SV) for each candidate RG. Different from geNorm, the NormFinder takes information by comparing the variation within and between user-defined sample groups, such as “Untreated/Treatment I/Treatment II, etc.” The SV is given by a combined measure of intra-and intergroup-variation associated to the candidate RGs expression. The lower SV, the more stable are the expressed RGs. The fundamental principle is that a stable candidate RG should have minimal variation across experimental groups and subgroups [9].

The BestKeeper [11] algorithm determines the most stable gene from a panel of up to ten potential candidate RGs. The geometric mean of the Cq values for each sample across all potential RGs are combined together to form the BestKeeper index. Then, each individual gene is compared in a pairwise fashion by Pearson correlation coefficient to the BestKeeper index (gene with the highest coefficient of correlation with the BestKeeper index indicates the highest stability and the highest ranked gene is the most stable). Pfaffl et al. [11] suggest the use of the best three to four most stable RGs to provide adequate normalization of the results.

The ΔCt method compares the relative expression of all pairwise combination of candidate RGs within each condition to identify which pairs show less variability and hence which gene(s) has the most stable expression by calculating the average SD of the relative expression of the pair of genes (the lower the average SD, the more stable the candidate RG expression) [12].

## MIQE guidelines

In the present work, the Minimum Information for Publication of Quantitative Real-Time PCR Experiments (MIQE) guidelines [5] was followed aiming experimental stringency and transparency, in order to increase the reliability and integrity of the data obtained **(**Additional file 4: Table S1).

## Results

### RGs and target transcripts: data mining and expression under abiotic stresses

All proposed candidate RGs present known functions/annotations (Table 1**)** and are involved in basal or vital cellular processes, as expected for a potential normalizer gene. The candidate RG “*UNK*” (XM_007131494.1; namely as “unknown” by Borges et al. [27]) was annotated in our work (through genetic ontology) as putative membrane protein (similar to AT3G13410; *Arabidopsis thaliana*). The initial screening of nine candidate RGs (except *ZMP* and *GAPC*) and four target transcripts (Table 1) by qPCR showed that all evaluated primer pairs were functional in all cowpea samples, amplifying a single band as indicated by the presence of a single peak in melting curves (Additional file 5: S2 Appendix). Means of Cq (quantification cycle) for each candidate RG varied from 16.58 (*VuUBQ10*) to 22.17 (*FBOX*), for root dehydration stress (Additional file 6: Table S2**)**, and from 15.58 (*VuUBQ10*) to 21.61 (*FBOX*), for salt stress (Additional file 7: Table S3). It is noteworthy that *VuUBQ10* and *FBOX* presented, respectively, the highest and smallest transcripts average abundance in both analyzed conditions. Considering, preliminarily, a stringent Cqs SD (standard deviation) < 1 associated to CVs (coefficient of variance), all potential RGs were constitutively expressed in the treatments evaluated, with the exception of *EF1*-*α* (11.36 ± 1.90), *β*-*TUB* (6.70 ± 1.33), and *VuUBQ10* (6.64 ± 1.04), concerning samples under salt stress (Table 2). However, *β*-*TUB*, *VuUBQ10*, and *EF1*-*α* were maintained in the study, in order to corroborate its expression using the more robust strategies.

**Table 2:** Coefficient of variance (CV) and standard deviation (SD) based on Cqs values of the candidates to cowpea reference genes

Gene	Salt stress (NaCl, 100 mM)	Root dehydration
	CV (%) ± SD	CV (%) ± SD
*UNK*	4.54 ± 0.82	3.57 ± 0.73
*β*-*TUB*	6.70 ± 1.33	3.37 ± 0.67
*FBOX*	4.23 ± 0.91	2.97 ± 0.66
*UE21D*	4.54 ± 0.84	3.24 ± 0.62
*VuUBQ10*	6.64 ± 1.04	3.17 ± 0.53
*Vu*ACT	4.40 ± 0.90	3.28 ± 0.71
*EF1*-*α*	11.36 ± 1.90	3.66 ± 0.62

*Vu* (*Vigna unguiculata*); *β*-*TUB* (beta-tubulin); *EF1*-*α* (elongation factor 1-alfa); *VuACT* (actin); *UE21D* (ubiquitin-conjugating enzyme E2 variant 1D); *UNK* (*Phaseolus vulgaris* unknown gene); *FBOX* (F-box protein); *VuUBQ10* (polyubiquitin 10)

The selection of target transcripts (*VuCHI*, chalcone isomerase; *VuCHS*, chalcone synthase; *VuLTP*, lipid transfer protein and *VuCHiB*, chitinase B) (Table 3) was based on their regulation in the HT-SuperSAGE libraries (see Methods) of the Cowpea Functional Genome Consortium (CpFGC). Despite their presence in both experiments, their regulation was distinct (Table 3). All target transcripts were up-regulated (UR) in ‘Pingo de Ouro’ (drought-tolerant accession), whereas in ‘Santo Inácio’ (drought-sensitive accession) their expression was variable including UR, down-regulation (DR) or not differential expression [also denominated not significant (ns) at the level of p ≤ 0.05] (Table 3). In turn, HT-SuperSAGE data for salt treatment indicated *VuLTP* and *VuCHS* as interesting target transcripts. While the *VuLTP* was UR in the ‘Pitiúba’ (salt-tolerant accession) and DR in the ‘BR14-Mulato’ (salt-sensitive accession); *VuCHS* was UR, in the salt-tolerant and “ns” in the salt-sensitive accession (Table 3).

**Table 3:** Transcriptional modulation of selected cowpea targets transcripts in the accessions and treatments analyzed

Gene	Unitag name	Root dehydration				Salt stress (NaCl, 100 mM)			
		Tolerant accession (Pingo de Ouro)		Sensitive accession (Santo Inácio)		Tolerant accession (Pitiúba)		Sensitive accession (BR14-Mulato)	
		FC	Reg. (*)	FC	Reg. (*)	FC	Reg. (*)	FC	Reg. (*)
*VuChiB*	Cp1020	5.10	UR	− 1.09	ns	1.20	ns	4.40	UR
*VuLTP*	Cp1050	11.21	UR	− 1.94	DR	2.80	UR	− 7.90	DR
*VuCHI*	Cp1131	5.65	UR	1.15	ns	1.20	ns	9.50	UR
*VuCHS*	Cp2022	47.70	UR	13.70	UR	2.20	UR	1.40	ns

Reg. (gene regulation); FC [Fold change: measure describing how much a quantity changes going from an initial (control) to a final value (treatment)]; UR: (up-regulated); DR: (down-regulated); ns (not significant at the level of p ≤ 0.05). *Vu* (*Vigna unguiculata*); *VuCHiB* (chitinase B); *VuLTP* (lipid transfer protein); *VuCHI* (chalcone isomerase); *VuCHS* (chalcone synthase)

*Gene regulation at the level of p ≤ 0.05

Considering the functional primer pairs, all amplification efficiency values in the qPCR analysis presented acceptable values (90 to 110% [28]) and ranged between 95.68 and 106.28% (Table 4). The y-intercept values varied from 33.12 to 38.03, while linear regression coefficients (*r*^*2*^) for all seven genes were ≥ 0.990 (Table 4).

**Table 4:** Characterization of cowpea qPCR reactions, indicating the category of the primer pairs used, amplified CRG or TT name, source (reference), efficiency (%) and sensitivity (y-intercept)

Category	Name	Reference	Slope (−)	Efficiency (%)	R^2^	y-intercept
CRG	*VuACT*	CpFGC	3.18	106.28	− 0.989	35.09
CRG	*β*-*TUB*	[25]	3.25	103.09	− 0.993	35.34
CRG	*EF1*-*α*	[26]	3.38	97.63	− 0.995	33.12
CRG	*FBOX*	[27]	3.28	101.78	− 0.982	35.77
CRG	*UE21D*	[27]	3.30	100.92	− 0.995	34.63
CRG	*VuUBQ10*	CpFGC	3.40	96.84	− 0.997	38.01
CRG	*UNK*	[27]	3.35	98.84	− 0.989	35.17
TT	*VuChiB*	CpFGC	3.40	96.84	− 0.996	38.03
TT	*VuLTP*	CpFGC	3.38	97.24	− 0.995	35.56
TT	*VuCHI*	CpFGC	3.43	95.68	− 0.996	37.01
TT	*VuCHS*	CpFGC	3.31	100.50	− 0.996	33.69

CRG (Candidate Reference Gene); TT (Target Transcript); Cowpea Functional Genome Consortium (CpFGC); *Vu* (*Vigna unguiculata*); *β*-*TUB* (beta-tubulin); *EF1*-*α* (elongation factor 1-alfa); *VuACT* (actin); *UE21D* (ubiquitin-conjugating enzyme E2 variant 1D); *UNK* (*Phaseolus vulgaris* unknown gene); *FBOX* (F-box protein); *VuUBQ10* (polyubiquitin 10); *VuCHiB* (chitinase B); *VuLTP* (lipid transfer protein); *VuCHI* (chalcone isomerase); *VuCHS* (chalcone synthase); R^2^ (Coefficient of Determination)

### Expression stability of candidates RGs based on four different statistical analyses

In the present study, the expression stability of the candidate RGs was analyzed in accessions under abiotic stress [root dehydration or salt (NaCl, 100 mM)], using four different approaches: geNorm, NormFinder, BestKeeper and ΔCt method.

**Fig. 1:**
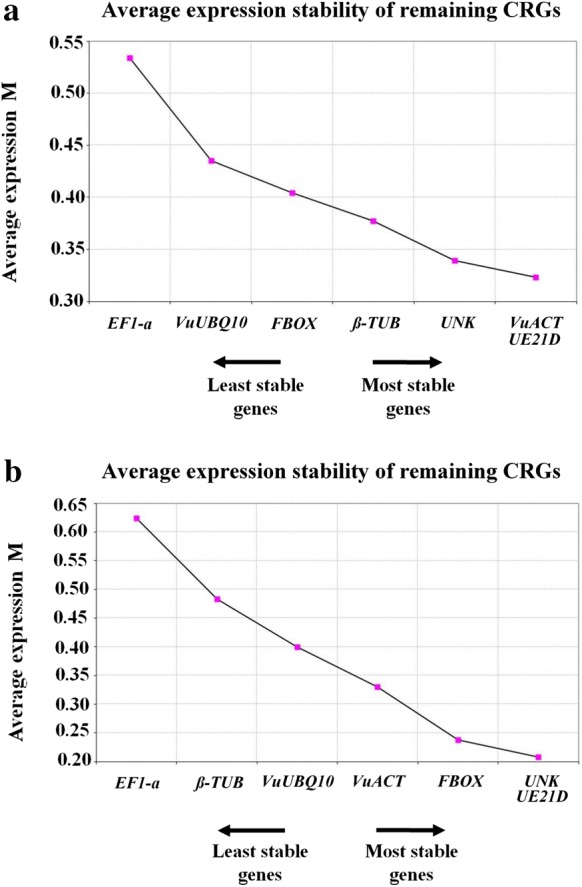
geNorm analysis, indicating the average expression stability (M value) of all seven candidate reference genes in cowpea accessions under: **a** root dehydration stress; and **b** salt stress (NaCl, 100 mM). The most stably expressed genes present lower M values. CRGs (Candidate Reference Genes); *Vu* (*Vigna unguiculata*); *β*-*TUB* (beta-tubulin); *EF1*-*α* (elongation factor 1-alfa); *VuACT* (actin); *UE21D* (ubiquitin-conjugating enzyme E2 variant 1D); *UNK* (*Phaseolus vulgaris* unknown gene); *FBOX* (F-box protein); *VuUBQ10* (polyubiquitin 10). *The two most stable RGs of the geNorm analysis cannot be ranked in order because of the required use of gene ratios for gene-stability measurements [10]

According to **GeNorm** analysis, all candidate RGs tested showed reduced “M” values (Fig. 1a, b), below 1.5, which is the default limit. Considering that the RGs are not co-regulated, stepwise exclusion of the gene with the highest “M” value brings a combination of two RGs that had the most stable expressions of the tested samples. For root dehydration, the most stable candidate RGs were *VuACT*/*UE21D*, followed by *UNK* and *β*-*TUB* (Fig. 1a). The two most stable RGs cannot be ranked in order because of the required use of gene ratios for gene stability measurements [10]. For salt stress treatment, after the stepwise exclusion of the gene with the highest “M” value, the four best RGs were *UNK*/*UE21D*, followed by *FBOX* and *VuACT* (Fig. 1b).

**Fig. 2:**
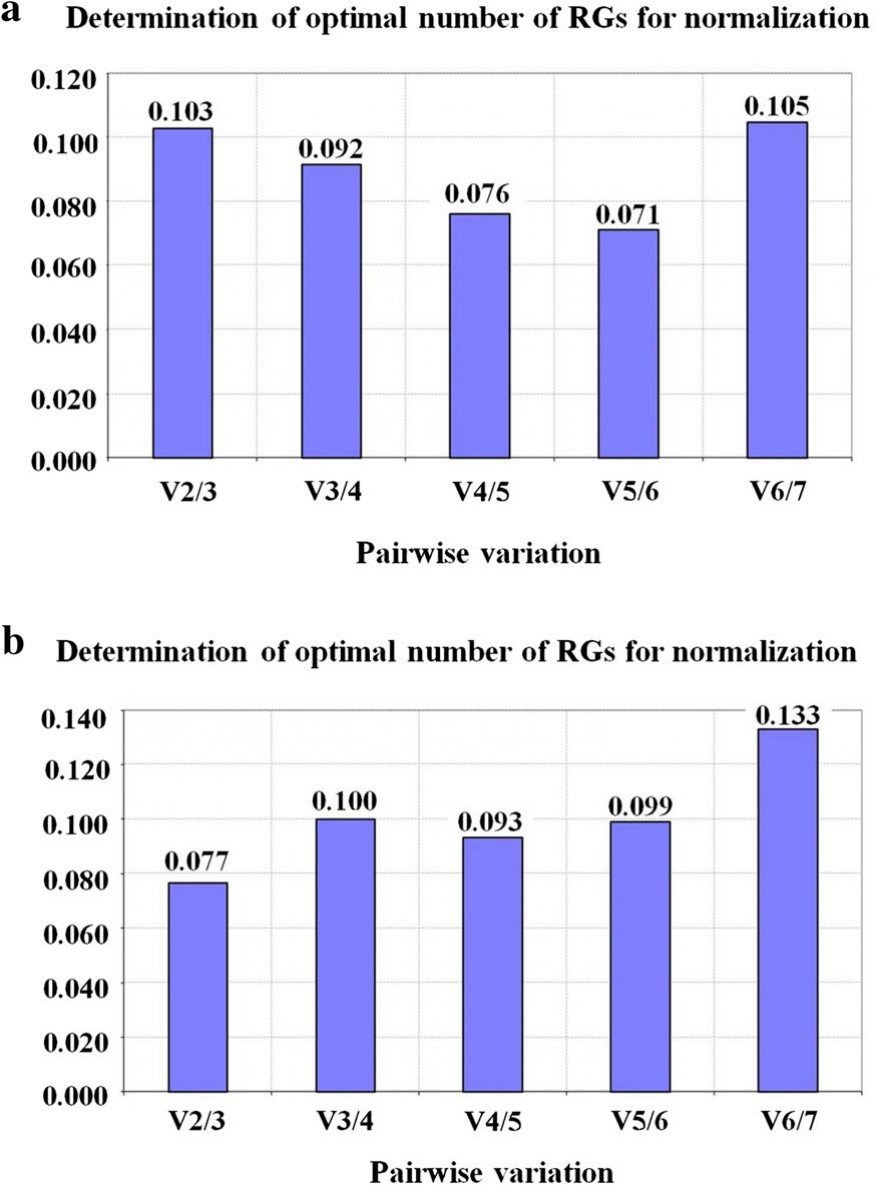
geNorm output, calculated by pairwise variation analysis between normalization factors NF_n_ and NF_n + 1_, indicating the optimal number of reference genes (RGs) required for reliable normalization in cowpea accessions under: **a** Root dehydration stress and **b** salt stress (NaCl, 100 mM). RGs (Reference Genes)

The geNorm also gives an estimate of the optimal number of RGs necessary for reliable normalization. This value is obtained from the “V” value analysis. A V-value below the established 0.15 threshold suggested by Vandesompele et al. [10] indicates that inclusion of an additional gene is not required for data normalization. Since this value was already reached after the first analysis (V2/3) for both assays (Fig. 2a, b), the inclusion of an additional candidate RG is not required. Thus, the two RGs could be used for normalization under these conditions; however, the use of the three most stable RGs for calculation of a qPCR normalization factor is recommended [10].

The best candidate reference genes according to **NormFinder** are those with the lowest stability value (Table 5), with minimal intra- and inter-group variation. Regard to root dehydration, the most stable were: *β*-*TUB* (0.104); *UE21D* (0.122); *FBOX* (0.138); and *VuACT* (0.141) (Table 5). For salt stress, the best candidate RGs were: *FBOX* (0.099); *UE21D* (0.116); *UNK*; and *β*-*TUB* (Table 5). *UNK* and *β*-*TUB* presented a stability value of 0.125 and assumed different positions for ranking purposes in Table 5.

**Table 5:** The NormFinder analysis of candidate reference genes (RGs) showing stability values in both experiments performed (a lower value indicates a more stable expression)

RGs acronym	Root Dehydration stress		Salt stress (NaCl, 100 mM)	
	Stability value	Rank	Stability value	Rank
*β*-*TUB*	0.104	1	0.125	4
*UE21D*	0.122	2	0.116	2
*FBOX*	0.138	3	0.099	1
*VuACT*	0.141	4	0.162	5
*UNK*	0.144	5	0.125	3
*VuUBQ10*	0.190	6	0.177	6
*EF1*-*α*	0.227	7	0.324	7

*Vu* (*Vigna unguiculata*); *β*-*TUB* (beta-tubulin); *UE21D* (ubiquitin-conjugating enzyme E2 variant 1D); *FBOX* (F-box protein); *VuACT* (Actin); *UNK* (*Phaseolus vulgaris* unknown gene); *VuUBQ10* (polyubiquitin 10) and *EF1*-*α* (elongation factor 1-alfa)

**Fig. 3:**
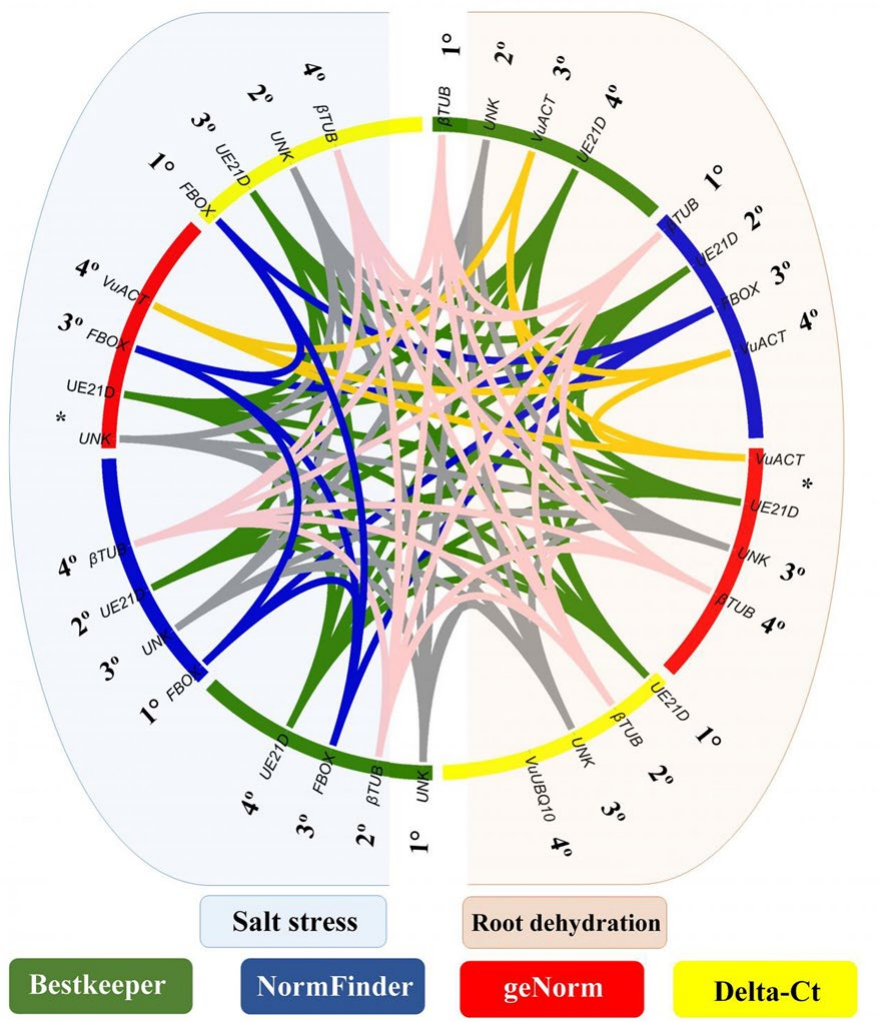
Intra- and inter-assay comparisons of the four most stable cowpea candidate reference genes, considering geNorm, NormFinder, BestKeeper, and ΔCt approaches. *Vu* (*Vigna unguiculata*); *β*-*TUB* (beta-tubulin); *EF1*-*α* (elongation factor 1-alfa); *VuACT* (actin); *UE21D* (ubiquitin-conjugating enzyme E2 variant 1D); *UNK* (*Phaseolus vulgaris* unknown gene); *FBOX* (F-box protein); *VuUBQ10* (polyubiquitin 10); *The two most stable candidate reference genes of this algorithm cannot be ranked in order because of the required use of gene ratios for gene-stability measurements [10]

The output of the NormFinder analysis revealed similar results to the geNorm. Both algorithms suggested *UNK*, *UE21D*, and *FBOX* as three of four most stable genes (Fig. 3) for salt stress; for root dehydration assay, the referred strategies listed *UE21D*, *VuACT*, and *β*-*TUB* among the four most stable (Fig. 3).

The **BestKeepe**r algorithm computes Pearson correlation coefficient to the BestKeeper index. The candidate reference gene with the highest Pearson coefficient of correlation with the BestKeeper index presents the highest stability. The analysis revealed *β*-*TUB* (0.958), *UNK* (0.925), *VuACT* (0.910), and *UE21D* (0.875; Table 6) as the four most stable RGs for root dehydration [following the results from geNorm (Fig. 3)]. Besides these, *β*-*TUB*, *UE21D*, and *VuACT* were also among the most stable, as indicated by NormFinder (Fig. 3).

**Table 6:** qPCR descriptive statistics of the candidate reference genes in cowpea under root dehydration and salt stress (NaCl, 100 mM), measured by BestKeeper software

Abiotic stress	Candidate reference gene	Rank	Pairwise correlation (PC) coefficient						Min	Max	SD	PC coefficient	*p* value
			GM	AM	min	max	SD	CV	[x-fold]	[x-fold]	[± x-fold]	[r]	
Salt stress	*UNK*	1	19.91	19.93	18.60	22.10	0.64	3.24	− 2.47	4.50	1.58	0.982	0.001
	*β*-*TUB*	2	19.81	19.85	18.00	23.40	1.00	5.03	− 3.60	12.71	2.03	0.979	0.001
	*FBOX*	3	21.59	21.61	20.10	24.10	0.68	3.13	− 2.85	5.84	1.62	0.975	0.001
	*UE21D*	4	18.40	18.42	17.00	20.70	0.60	3.24	− 2.65	4.99	1.52	0.975	0.001
	*VuUBQ10*	5	15.55	15.58	13.80	18.00	0.78	5.03	− 3.27	5.28	1.74	0.920	0.001
	*VuACT*	6	20.41	20.43	18.70	23.00	0.66	3.23	− 3.44	6.50	1.59	0.915	0.001
	*EF1*-α*	7	16.58	16.68	14.40	22.80	1.36	8.16	− 4.43	70.08	2.62	0.948	0.001
Root dehydration	*β*-*TUB*	1	19.93	19.95	18.60	21.30	0.55	2.76	− 2.57	2.63	1.48	0.958	0.001
	*UNK*	2	20.35	20.37	19.30	22.20	0.60	2.95	− 2.07	3.56	1.53	0.925	0.001
	*VuACT*	3	21.74	21.75	20.60	23.50	0.53	2.45	− 2.28	3.56	1.46	0.910	0.001
	*UE21D*	4	19.04	19.05	18.07	20.40	0.51	2.65	− 1.97	2.59	1.43	0.875	0.001
	*FBOX*	5	22.16	22.17	21.20	23.70	0.56	2.52	− 1.96	2.96	1.48	0.870	0.001
	*VuUBQ10*	6	16.57	16.58	15.80	17.90	0.45	2.70	− 1.68	2.47	1.37	0.816	0.001
	*EF1*-*α*	7	16.84	16.85	15.80	18.50	0.47	2.78	− 2.03	3.11	1.39	0.475	0.019

*Vu* (*Vigna unguiculata*); *UNK* (*Phaseolus vulgaris* unknown gene); *β*-*TUB* (beta-tubulin); *FBOX* (F-box protein); *UE21D* (ubiquitin-conjugating enzyme E2 variant 1D); *VuUBQ10* (polyubiquitin 10); *VuACT* (actin); *EF1*-*α* (elongation Factor 1-alfa); GM: the geometric mean of PC; AM: the arithmetic mean of PC; Min PC and Max PC: the extreme values of PC; SD: the standard deviation of the PC; CV: the coefficient of variance expressed as a percentage of the PC level; Min [x-fold] and Max [x-fold]: the extreme values of expression levels expressed as an absolute x-fold over- or under-regulation coefficient; SD [± x-fold]: standard deviation of the absolute regulation coefficient; [r]: Pearson correlation coefficient

*Inconsistent data: SD > 1 [11]

For salt stress, the four most stable RGs were *UNK* (0.982), *β*-*TUB* (0.979), *FBOX* and *UE21D* (both 0.975) (Table 6). *FBOX* and *UE21D* exhibited identical “r” values (despite presenting different positions in Table 6 due to their ranking). The four most stable candidate RGs indicated by BestKeeper were the same as those indicated by NormFinder (Fig. 3); while in geNorm, *UNK* and *FBOX* and *UE21D* also figured among the four most stable (Fig. 3).

**Fig. 4:**
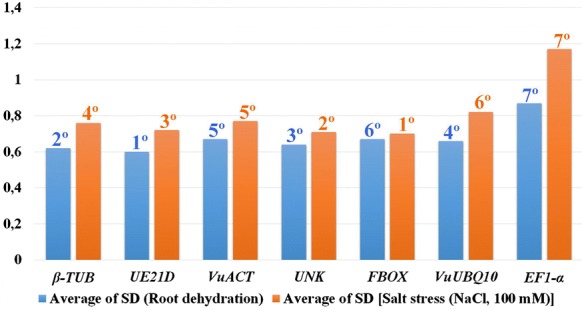
Cowpea candidate reference genes evaluated by the ΔCt method and ranked according to expression stability for root dehydration and salt assays. *Vu* (*Vigna unguiculata*); *β*-*TUB* (beta-tubulin); *UE21D* (ubiquitin-conjugating enzyme E2 variant 1D); *VuACT* (actin); *UNK* (*Phaseolus vulgaris* unknown gene); *FBOX* (F-box protein); *VuUBQ10* (polyubiquitin 10) and *EF1*-*α* (elongation factor 1-alfa)

The **ΔCt method** is based on the comparison of ‘pairs of genes’ using a simple ΔCt approach. All pairs of candidate reference genes are compared to each other, and the genes are ranked according to the average standard deviation (SD) based on the relative expression of the pair of genes (the lower the average SD, the more stable is the candidate RG). For root dehydration, the most stable were, respectively: *UE21D* (0.60), *β*-*TUB* (0.62), *UNK* (0.64), and *VuUBQ10* (0.66) (Fig. 4). This set contains 75% of RGs also ranked as more stable by geNorm and BestKeeper (Fig. 3); compared to NormFinder this result was 50% (Fig. 3). For salt treatment, the most stable, respectively, were *FBOX* (0.70), *UNK* (0.71), *UE21D* (0.72), and *β*-*TUB* (0.76) (Fig. 4). Again, a confluence of 75% of the data was observed qualitatively in relation to the other approaches used (Fig. 3).

Considering both stresses analyzed, the gene *EF1*-*α* was the less stable candidate RG, as also revealed by geNorm, NormFinder and Bestkeeper (Figs. 1a, b, 4; Tables 5, 6).

### Conservation of the candidates RGs stability between both abiotic stresses

Comparing the results, based on the data of the two analyzed assays and the algorithms employed, including the ΔCt method, some of the RG candidates presented as the most stable on one stress condition were also considered stable in the other stressful situation. Among the candidate RGs showing this expression stability, it is worth mentioning:

- *UE21D*, which is one of the four most stable during salt stress assay, considering the four analytical approaches; and one the two most stable during the root dehydration assay, considering the geNorm, NormFinder, and ΔCt strategies (Fig. 3);
- *UNK*: it was considered among the three most stable during the salt stress assay (based on the four analytical approaches), and also among the three most stable during the root dehydration assay based on the geNorm, BestKeeper and ΔCt approaches (Fig. 3).

### Reference genes choice and HT-SuperSAGE data validation by qPCR

Considering that HT-SuperSAGE and qPCR are different approaches, the expression levels in both methods are not expected to be identical. So, to validate the HT-SuperSAGE data, the samples were not pooled for the qPCR analysis (as they were for HT-SuperSAGE libraries analysis; see Methods section). An agreement between both approaches was considered when, at least in one-time point, similar gene expression regulation was demonstrated in both approaches (HT-SuperSAGE and qPCR), as adopted by Ferreira Neto et al. [31].

The most stable genes under both stresses suggested by the geNorm (which were also the most stable by the others algorithms) (Fig. 3) were used as reference genes for the expression validation of the HT-SuperSAGE data. Thus, *VuACT*, *UE21D*, and *UNK* (Fig. 1a) were the selected RGs for validation of the root dehydration assay, whereas *UNK*, *UE21D*, and *FBOX* (Fig. 1b) were chosen for salt stress assay.

Concerning the root dehydration qPCR assay, the target *VuLTP* was up-regulated in the drought-tolerant accession (Pingo de Ouro) only 150 min after the stress imposition (Table 7; Additional file 8: Table S4). In turn, in relation to the drought-sensitive accession (Santo Inácio), this target showed no differential expression (ns) during all exposition times (Table 7; Additional file 8: Table S4). Considering the salt stress assay, *VuLTP* also presented up-regulation by the salt-tolerant accession (Pitiúba) at all the analyzed stress times (Table 7; Additional file 9: Table S5), opposite to the salt-sensitive accession (BR14-Mulato), which showed no differential expression in the referred time points (Table 7; Additional file 9: Table S5).

**Table 7:** Comparison between the HT-SuperSAGE expression libraries and qPCR data in cowpea roots under abiotic stress treatments

Gene	Root dehydration										Salt stress (NaCl, 100 mM)									
	HT-SS assay†		qPCR assay*								HT-SS assay†		qPCR assay*							
			(Pingo de Ouro) TOL			VGEǂ	(Santo Inácio) SEN			VGEǂ			(Pitiúba) TOL			VGEǂ	(BR14-Mulato) SEN			VGEǂ
	TOL	SEN	25′	75′	150′		25′	75′	150′		TOL	SEN	30′	60′	90′		30′	60′	90′	
*VuChiB*	UR	ns	ns	ns	ns	No	UR	ns	ns	Yes	ns	UR	ns	ns	UR	Yes	DR	DR	ns	No
*VuLTP*	UR	DR	ns	ns	UR	Yes	ns	ns	ns	No	UR	DR	UR	UR	UR	Yes	ns	ns	ns	No
*VuCHI*	UR	UR	ns	UR	UR	Yes	UR	UR	UR	Yes	UR	ns	UR	UR	UR	Yes	UR	UR	UR	No
*VuCHS*	UR	ns	UR	UR	UR	Yes	UR	UR	UR	No	ns	UR	UR	UR	UR	No	UR	UR	ns	Yes

HT-SS (HT-SuperSAGE); *Vu* (*Vigna unguiculata*); *VuCHiB* (Chitinase B); *VuLTP* (Lipid transfer protein); *VuCHS* (Chalcone synthase); *VuCHI* (Chalcone isomerase)

† and * p < 0.05. TOL (tolerant accession); SEN (sensitive accession); UR (up-regulated); DR (down-regulated); ns (not significant at p < 0.05); 25′, 75′ and 150′ (minutes under root dehydration); 30′, 60′ and 90′ (minutes under salt stress), VGE (Validation of Gene Expression); **ǂ** between HT-SuperSAGE and qPCR data

The contrasting drought-responsive accessions differed regarding the expression level of *VuCHiB*, in the course of the qPCR-tested times. Santo Inácio showed up-regulation only at the first time point (25 min). However, the expression of *VuCHiB* did not change at any time in Pingo de Ouro (Table 7; Additional file 8: Table S4). Considering the salt stress, Pitiúba showed up-regulation only at the last time (90 min), while BR14-Mulato showed down-regulation in the first two times (Table 7; Additional file 9: Table S5). *VuCHiB* was the only down-regulated transcript from the stress imposition analyzed by qPCR.

qPCR analysis indicated that the *VuCHS* gene was up-regulated in both accessions under root dehydration over all the stressful times (Table 7; Additional file 8: Table S4). For salt stress, the same transcript was up-regulated in ‘Pitiúba’ in 30, 60, and 90 min; while BR14-Mulato showed up-regulation in 30 and 60 min (Table 7; Additional file 9: Table S5).

The up-regulation of *VuCHI* transcript in cowpea Pingo de Ouro accession was confirmed by qPCR at 75 and 150 min. However, contrary to results from HT-SuperSAGE, qPCR data showed that the Santo Inácio accession was up-regulated in all the analyzed times after stress (Table 7; Additional file 8: Table S4). In salt stress assay, *VuCHI* was also up-regulated in both Pitiúba and BR-14 Mulato accessions, in all the stress times (Table 7; Additional file 9: Table S5).

The results of qPCR and expression libraries were validated for nine of sixteen comparisons (approximately 56%) (Table 7). In addition, qPCR data suggests *VuLTP*, *VuCHI*, and *VuCHS* (in both abiotic stresses studied) as potential targets for biotechnological approaches. This is due to two findings: (1) *VuLTP* is up-regulated in both tolerant accessions and has different regulation on both respective sensitive accessions, for both stress conditions studied; (2) the results indicate the participation (up-regulation) of the *VuCHI* and *VuCHS* in response to the abiotic stresses analyzed, even in cowpea accessions quite genetically distinct (tolerant and sensitive).

On the other hand, *VuCHiB* presents biotechnological potential regarding salt stress, only. The tolerant accession up-regulated a transcript coding the referred enzyme, while sensitive accession presented down-regulation of this target (Table 7; Additional file 9: Table S5).

## Discussion

Due to the existence of potential errors during the preparation, synthesis, sequencing, and analysis of transcriptomic libraries (e.g., subtractive libraries, HT-SuperSAGE, RNA-Seq, among others), a second technique is required to validate (corroborate) the gene expression results. The currently most used technique for such purpose is quantitative real-time PCR [32], considered a gold standard validation method. Therefore, in order to ensure the reliability and precision of qPCR data, the MIQE guidelines [5] was applied for the acquisition of the results presented here (Additional file 4: Table S1). There is a lack of a systematic validation of RGs in cowpea (i.e., out of five works [13–17] addressing this theme, four omitted any information on how RGs expression stability and primer efficiency were evaluated). The present work represents a pioneering effort for a detailed analysis of candidate RGs in tolerant and sensitive cowpea cultivars in response to abiotic stresses (root dehydration and salt) rigorously tested for effective normalization of the qPCR data. These RGs may be also useful in the qPCR analysis of gene expression studies in other closely related species.

In terms of standardization and quality, all qPCR reactions (Table 4) showed amplification efficiencies between 90 and 110%, considered, therefore, acceptable [28]. According to Pfaffl et al. [29], uncorrected small efficiency differences between target and reference genes generate false expression ratio, resulting in over/under-estimation of the ‘real’ initial RNA amount. The y-intercept (33.12 to 38.03) and *r*^*2*^ (≥ 0.990) values (Table 4), for all seven RG candidates, revealed that the adopted setup for qPCR was also sufficient to obtain good efficiency, accuracy, and sensitivity [33].

Previous studies have reported the importance of using more than one statistical method for reference gene stability evaluation. Thus, it is expected that the comparison using different approaches might provide a more reliable set of RGs under a given experimental condition. Based on this, here we applied the four most common approaches (geNorm, NormFinder, Bestkeeper and the ΔCt method) to evaluate a set of candidate RGs for qPCR normalization of cowpea accessions after the abiotic stresses (root dehydration or salt) application. Intra-assay, for salt stress, there was a convergence of at least 75% in the genes indicated as most stable RGs, in regard to the four most stable suggested by all strategies (Fig. 3). For root dehydration, this value has been reached considering geNorm, BestKeeper, and NormFinder (Fig. 3). The variation occurred especially regarding their ranking (Fig. 3). Such discrepancies in ranking are not surprising, has been reported in previous studies [e.g., 34–36].

Considering its fundamental role in the protein biosynthesis [37], the housekeeping gene *EF1*-*α* has been used for normalization of qPCR data in some crop species, such as *Vigna mungo* [38], coffee [39] and potato [40], during salt stress. In the legume crop *Caragana intermedia* under osmotic, salt, cold and heat stress, the *EF1*-*α* gene showed to be stable using geNorm, NormFinder, and BestKeeper algorithms [41]. Also, in combination with *SAND* (SAND family protein) and *UNK2* (hypothetical protein), *EF1*-*α* was appropriate for normalizing gene expression data in salt-treated and in cold-treated leaves of the same species [41]. Contrarily, in our study, *EF1*-*α* gene was found to be one of the least stable, indicating that it is not a suitable reference gene in cowpea under root dehydration or salinity stress. Although housekeeping genes are generally indicated as good normalizers in qPCR data, these contrasting results have shown that they need to be evaluated efficiently in different species, tissues, and stress conditions.

In addition to *EF1*-*α,* actin and tubulin genes (both evolved in basic and essential processes in the cell) are also known as traditional RGs in plants [42]. *β*-*TUB* was ranked among the most stable genes considering the root dehydration samples and the applied strategies (Fig. 3), while *VuACT* was recommended using the geNorm, NormFinder, and Bestkeeper strategies (Fig. 3). Similarly, paralogous of actin (i.e., *ACT*-*1, 2, 4* or *11*) were ranked as the top most stable genes in cotton (for different stages of development of flower verticils and fruit) [43], in rice under salt stress [44], and in peanut under biotic and abiotic stresses [45].

Another candidate RG whose expression stability deserves mentioning codify a *UE21D* (Ubiquitin-conjugating enzyme E2 variant 1D). *UE21D* carries out the transfer of ubiquitin to a protein substrate and figures among the main enzymes in the regulatory step for the selective protein degradation mechanism. It is a crucial regulatory step for an essential housekeeping role by removing abnormal proteins that arise through biosynthetic errors and natural proteins that acquire non-native conformations, supplying amino acids needed to produce new proteins [46]. All applied methods indicated the *UE21D* among the four most stable for both stress types (Fig. 3). Thus, this candidate RG is indicated as a major actor for the standardization of qPCR reactions in cowpea root tissue under abiotic stresses, in contrast to its reduced expression stability previously observed by Borges et al. [27] in common bean (*Phaseolus vulgaris*) leaves under fungal infection (*Colletotrichum lindemuthianum*).

The difference between gene expression stability of *UE21D* and *VuUBQ10* is worth mentioning. As previously mentioned, *UE21D* was among the four most stable gene applying the four evaluated approaches, concerning both abiotic stresses studied (Fig. 3). In turn, *VuUBQ10* (also associated with ubiquitination processes) fell outside the list of the four most stable gene, in both analyzed situations (except for root dehydration, using the ΔCt method; Fig. 3). The fact that both genes are involved in ubiquitination mechanisms could, a priori, indicate a possible co-regulation, with close results in the rankings, reducing the reliability of our results. However, it was observed in Arabidopsis that even within the polyubiquitin group (divided in *UBQ3/UBQ4* and the *UBQ10*/*UBQ11/UBQ14* subtypes) the RNA level of their constituents are independently modulated, within and between subtypes [47]. One major limitation in geNorm is its insensitivity to coregulated candidate RGs, therefore demanding the choice of candidates preferentially from different pathways and functional classes [10]. However, it is sometimes difficult to avoid using coregulated genes for geNorm, especially when dealing with unknown, hypothetical or poorly annotated genes [9]. In this context, it is noteworthy that the supposed molecular function of *UNK*, based on the genetic ontology, is a membrane protein (Table 1), with no functional overlap with another candidate RGs analyzed here. When coregulated genes are absent, geNorm and NormFinder usually provide almost the same general ranking, with only minor differences in order, as observed in the present work (Fig. 3).

Another interesting result was that two candidate RGs (*UE21D* and *UNK*) considered among the most stable in root tissue under dehydration were also stable in the same tissue under salt stress (Fig. 3). It is known that plants exhibit a range number of response to environmental changes and that molecular mechanisms include signal recognition, signal transduction, and signal responses, among others [48]. Some of these mechanisms are shared among distinct stresses since plants are often exposed to a myriad of abiotic and biotic stresses under field conditions [48]. Thus, such RGs become strong candidates for application of stability tests on other abiotic stress types, with emphasis on those that have a similar physiological impact on tissues, like freezing, that imposes osmotic stress on plants, similarly to what occurs with drought and salt stresses [49].

The RG *F*-*BOX*, in turn, presented as one of the three most stable genes using the performed strategies and considering the salt stress applied. However, similar behavior was not evidenced (at least comprising the top four) considering the root dehydration stress samples (except by the NormFinder result; Table 5). Despite this, for the referred assay, the observed stability indices of this RG are within the acceptable standards indicated by the four approaches (stability value, NormFinder [9]; M-value, geNorm [10]; coefficient of correlation with the BestKeeper index [11]; and average SD for relative expression of ‘pairs of genes’; ΔCt approach [12]). Therefore, *F*-*BOX* is another important RG to be considered in gene expression of cowpea addressing saline or other osmotic stress.

Regarding the target transcripts, only the expression of the *VuCHI* gene was validated in both accessions under root dehydration (Table 7; Additional file 8: Table S4). The *VuCHI* (EC 5.5.1.6) is an enzyme of the isoflavonoid pathway in plants and catalyzes the cyclization of chalcone into (2S)-naringenin. Naringenin defines a critical branch point for the synthesis of several major classes of flavonoids, including flavanones, flavonols, and anthocyanins [50, 51]. Flavonoids have a significant contribution to the response mechanisms of higher plants to a variety of abiotic stresses. Its function is mainly associated with the inhibition of cellular reactive oxygen species (ROS) production [52]. Even considering the validation index of approximately 56%, it is important to emphasize that qPCR analyses indicated that these targets have biotechnological potential.

Besides *CHI*, also the gene encoding *VuCHS* presented up-regulation in both situations, and accessions studied, differently than the HT-SuperSAGE data indicated (Table 7). *VuCHS* (EC 2.3.1.74) is another key enzyme involved in the regulation of flavonoids biosynthesis. *VuCHS* is the entry point of the flavonoid pathway and catalyzes the transformation of the 4-Coumaroyl-CoA and Malonyl-CoA to chalcone, leading phenylpropanoids pathway to flavonoids biosynthesis [50, 51]. Thus, there are indications that compounds derived from the enzymatic action of *VuCHS* and *VuCHI* actively participate in the process of tolerance to stresses that cause an osmotic imbalance (such as root dehydration and salt stress) in cowpea.

The gene codifying a *VuLTP*, in turn, presented contrasting regulation between the accessions (Table 7; Additional File 8: Table S4; Additional File 9: Table S5), considering both conditions analyzed by qPCR. *VuLTP* gene was induced in the two tolerant employed accessions, while presented no differential expression in the sensitive counterparts. The involvement of this gene in response to drought in other plant species has been reported. Guo et al. [53] found that the rice loss-of-function mutant *LTP3* was sensitive to drought stress, whereas overexpressing plants were drought tolerant. Additionally, Wang et al. [54] observed that a wheat lipid transfer protein 3 (w*LTP*3) could enhance the basal thermotolerance and oxidative stress resistance in Arabidopsis.

The only transcript analyzed whose qPCR indicated down-regulation was *VuCHiB*, in the salt-sensitive accession. With regard to the salt-tolerant accession, *VuCHiB* was up-regulated in the last time point analyzed (90 min; Table 7; Additional File 9: Table S5). Chitinases are enzymes that degrade chitin (a linear polymer of β-1, 4-N-acetylglucosamine). Chitin is the second most abundant biopolymer on the planet [55] and is found in the outer skeleton of many organisms (insects, algae, yeasts, crabs, fungi, among other [56]). Plant chitinases are classified as PR (pathogen-related) proteins that act in plant self-defense against phytopathogens and pests [57]. Some chitinases genes are up-regulated in response to abiotic stresses, such as drought in cucumber [58] and high salt, in rice [53]. This fact, now, is also reported in cowpea tolerant accessions under radicular dehydration and salt stresses. Data mining in the CpFGC datasets uncovered just over a dozen *VuCHiB* isoforms (data not shown).

## Conclusion

Taken together, the here applied approaches allowed the identification of converging RGs within and between trials, considering the four different cowpea accessions analyzed. The presenting work represents the first evaluation of RGs for cowpea subjected to root dehydration or salt stress. Except for *EF1*-*α* (using BestKeeper algorithm for salt stress assay), all other candidates showed acceptable stability thresholds according to the four strategies. For root dehydration stress, the candidates *VuACT*, *UE21D*, used in qPCR validation activities, were ranked among the most stable genes in the four approaches scrutinized. The *UNK* gene also applied in the qPCR analysis with the root dehydration samples ranked among the most stable RGs considering three approaches (geNorm, BestKeeper, and ΔCt method). In turn, the candidates *UNK*, *UE21D*, and *FBOX* were the most stable genes for salt stress, based on all used strategies. In summary, these findings provide useful tools for the normalization of qPCR experiments and enable accurate and reliable gene expression evaluations related to cowpea transcriptomics.

Regarding the comparative analysis of the target genes, both qPCR and HT-SuperSAGE approaches presented a 56% agreement on results. This data may reflect a random sampling deviation or real technique variation, demonstrating the importance of validation of gene expression results in transcriptomic studies. The target genes represent promising candidates for biotechnological use after these validations. Our results suggest that flavonoids (or its derivatives) are essential players in the cowpea response to the conditions analyzed. This fact was observed from the up-regulation, in all accessions and situations, of transcripts encoding *VuCHS* and *VuCHI*, key enzymes in the synthesis of those compounds. According to qPCR analyses, *VuLTP*s also participate in the tolerance processes. This gene presented contrasting regulation between the accessions in the analyzed situations. The analyzed *VuLTP* was up-regulated in the tolerant ones, and no differentially expressed in the sensitive counterparts, for both stresses. Since these proteins are involved in multiple actions, their specific involvement or function still needs to be scrutinized. *VuCHiB*, in turn, is an interesting target gene only in cowpea under salt stress, due to its differential expression in both analyzed accessions (up-regulated in the tolerant; down-regulated in the sensitive). Such a target is commonly associated with plant responses to pathogenic organisms, adding value to its biotechnological potential since this gene can act in diverse situations.

Besides, cowpea beans, when compared to other legumes of economic importance, are still a crop with limited gene expression research. The present study provides some grounds for future research on the gene expression of cowpea under root dehydration or saline stress, or similar conditions that impose osmotic imbalance. The analyzed targets, in turn, aggregate information on the molecular physiology of cowpea under unfavorable conditions, as well as revealing a potential for future use in breeding programs.

## Additional files

**Additional file 1. Figure S1A and S1B.** Experimental design for the assays [radicular dehydration and salt (NaCl, 100 mM) presented in this work. Dashed lines represent the bulks formation to the HT-SuperSAGE libraries synthesis.

**Additional file 2. Appendix S1.** Sequences used in the present work.

**Additional file 3. Figure S2.** Schematic representation of the steps performed to analyze the candidate reference genes evaluated in the present study. **Legend**: SD (Standard Deviation); r^2^ (Pearson´s Correlation Coefficient); CRGs (Candidate Reference Genes); CpFGC (Cowpea Functional Genome Consortium).

**Additional file 4. Table S1.** MIQE checklist for reviewers, and editors. All essential information (E) must be submitted with the manuscript. Desirable information (D) should be submitted if available.

**Additional file 5. Appendix S2.** Melting curve for seven candidate reference genes [(*FBOX*) F-box protein; (*VuACT*) actin; (*VuUBQ10*) polyubiquitin 10; (*eEF*-*1α*) eukaryotic elongation factor 1α; (*β*-*TUB*) beta-tubulin; (*UNK*) *Phaseolus vulgaris* unknown gene; (*UE21D*) ubiquitin-conjugating enzyme E2 variant 1D] with single peak.

**Additional file 6. Table S2.** Cqs obtained for the candidate reference genes analyzed for the root dehydration assay.

**Additional file 7. Table S3.** Cqs obtained for the candidate reference genes analyzed for the salt stress assay (NaCl, 100 mM).

**Additional file 8. Table S4.** Data explored by REST software to analyze the relative expression of target transcripts in cowpea under root dehydration stress.

**Additional file 9. Table S5.** Data explored by REST software to analyze the relative expression of target transcripts in cowpea under salt stress.
