# Supplementary figures and images for: Cowpea and abiotic stresses: identification of reference genes for transcriptional profiling by qPCR
Source: Plant Methods. 2018 Oct 12;14:88. doi: 10.1186/s13007-018-0354-z (PMC6182843; doi:10.1186/s13007-018-0354-z)

**A**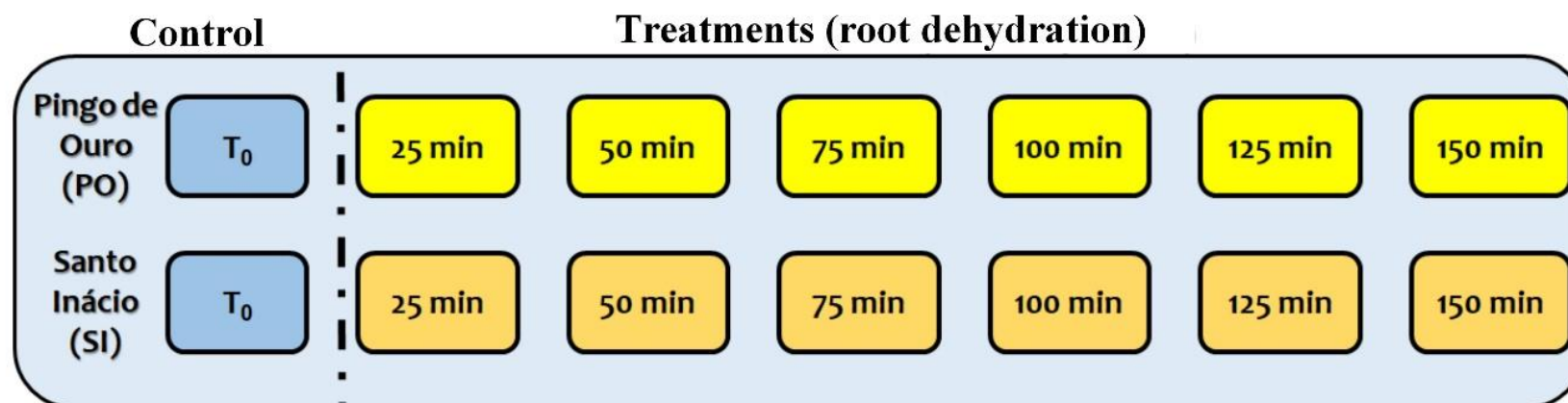**B**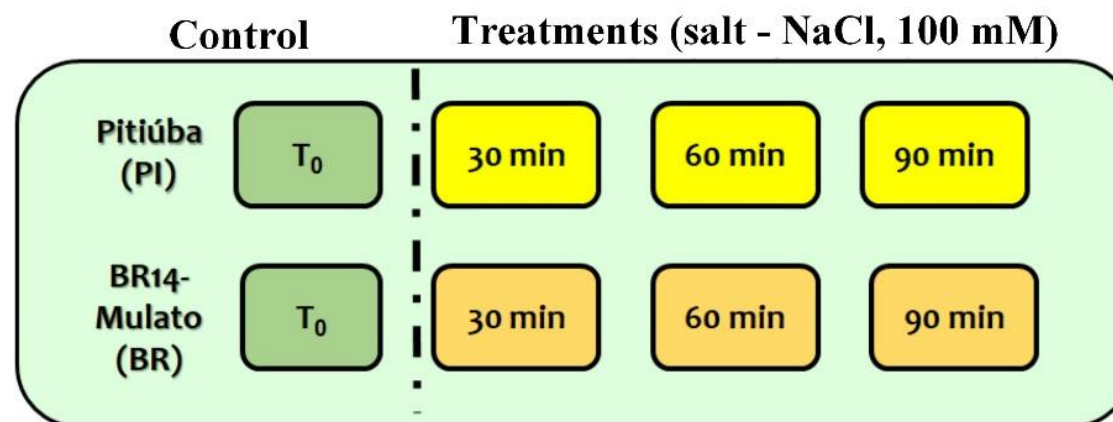

Supplement: Supplementary file 1 — Additional file 1. Figure S1A and S1B. Experimental design for the assays [radicular dehydration and salt (NaCl, 100 mM) presented in this work. Dashed lines represent the bulks formation to the HT-SuperSAGE libraries synthesis. [file 13007_2018_354_MOESM1_ESM.pdf]

## CRGs mining: CpFGC database and scientific literature

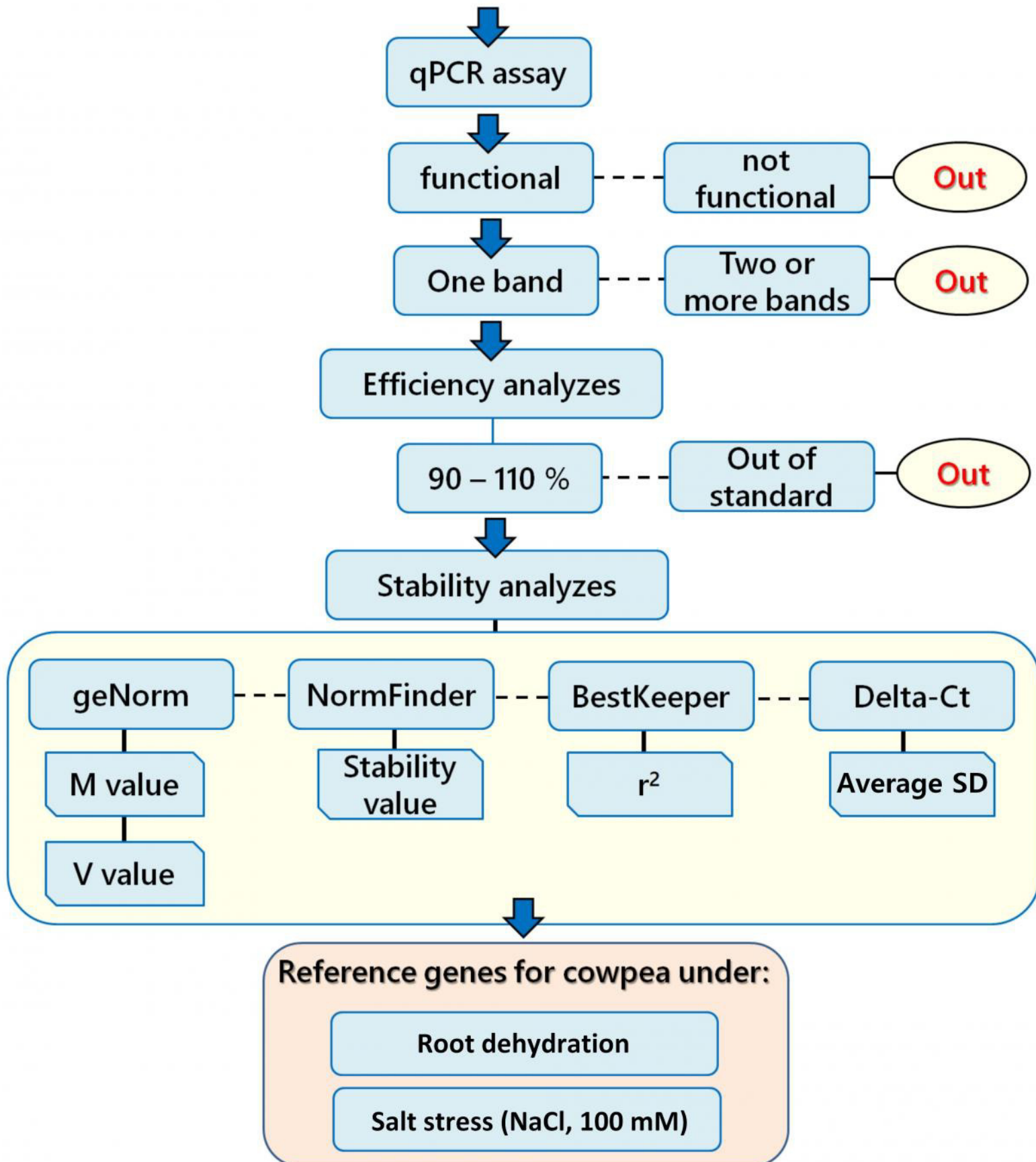

Supplement: Supplementary file 3 — Additional file 3. Figure S2. Schematic representation of the steps performed to analyze the candidate reference genes evaluated in the present study. Legend: SD (Standard Deviation); r2 (Pearson´s Correlation Coefficient); CRGs (Candidate Reference Genes); CpFGC (Cowpea Functional Genome Consortium). [file 13007_2018_354_MOESM3_ESM.pdf]

***FBOX***

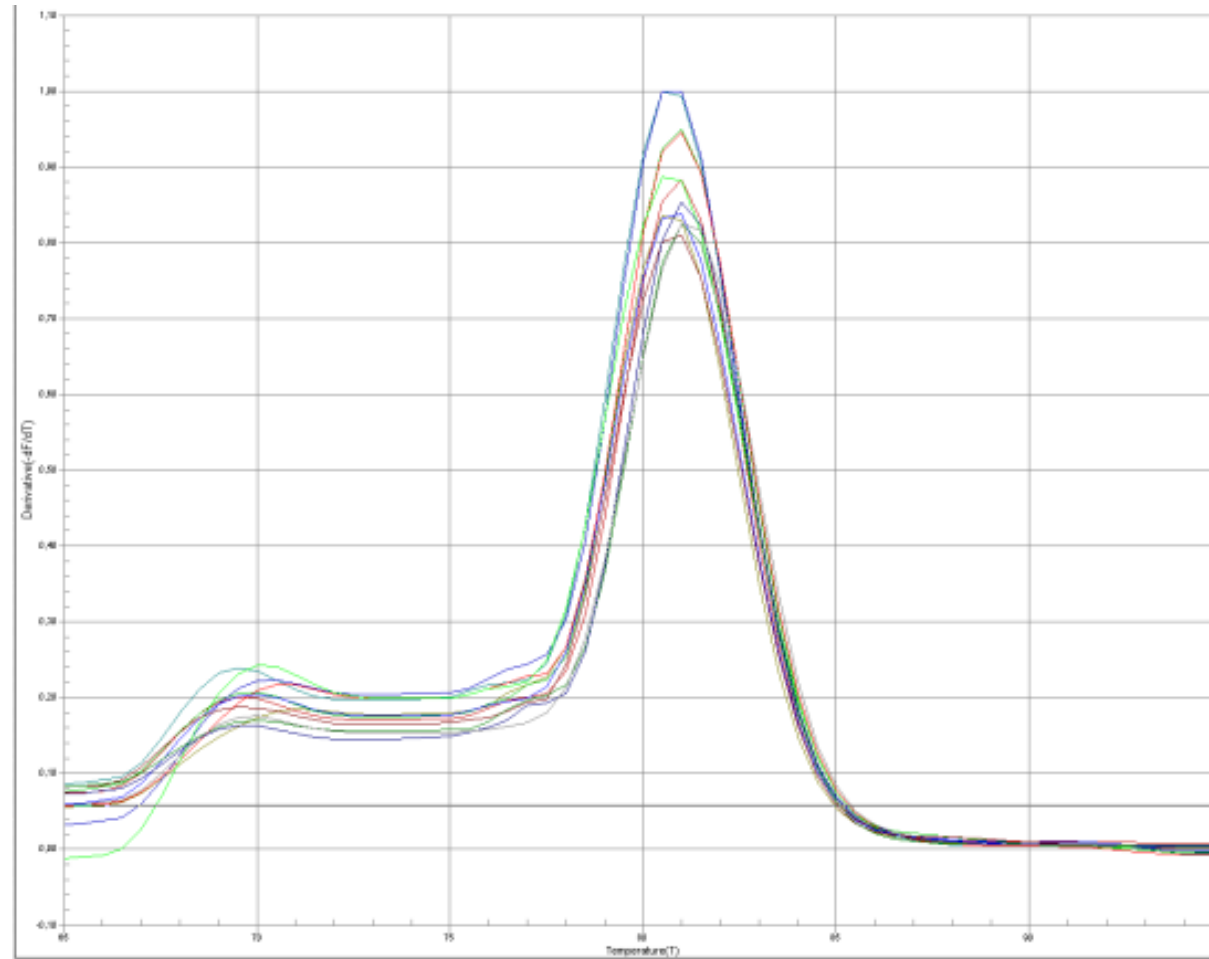

***VuACT***

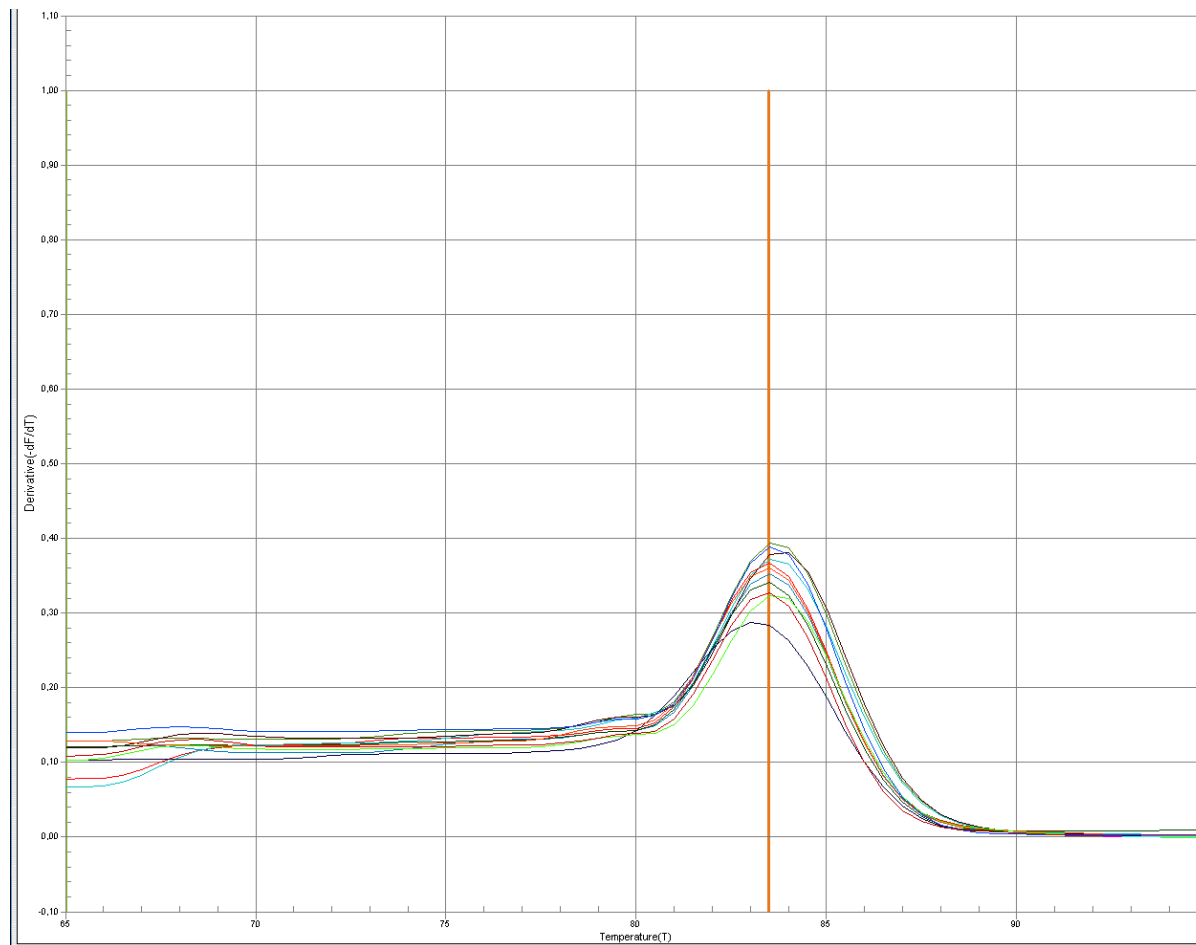

## *VuUBQ10*

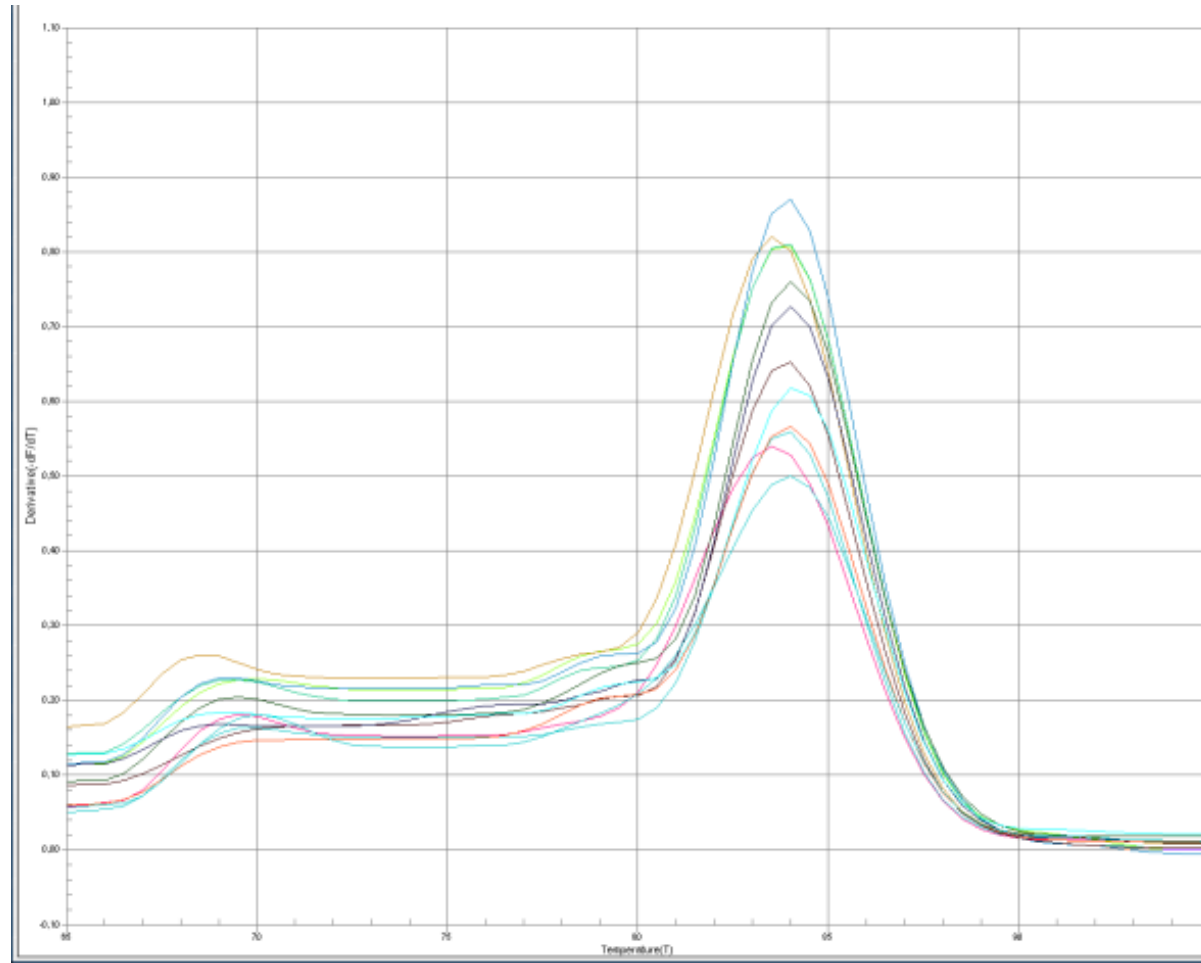

## ***$\beta$ -TUB***

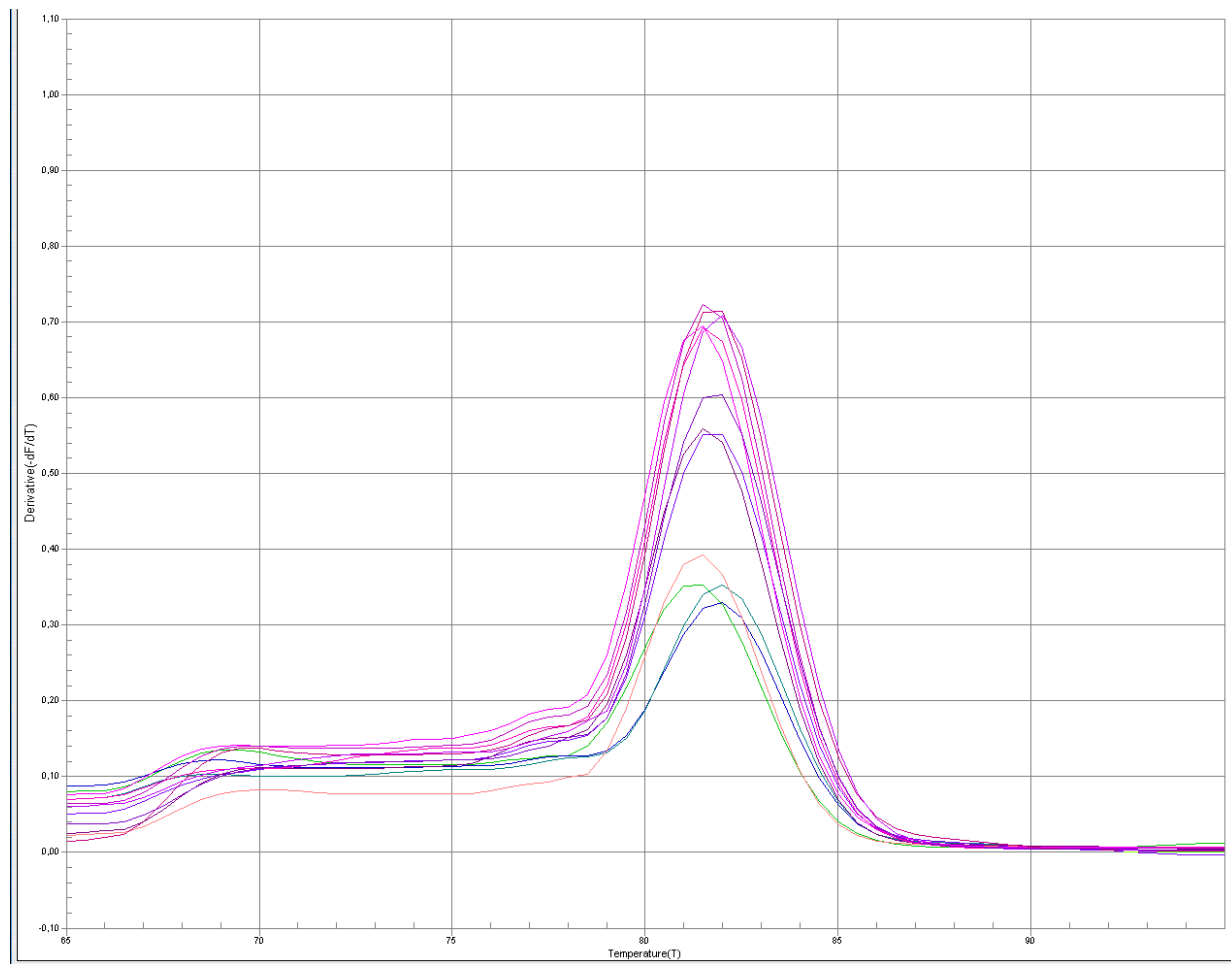

**UNK**

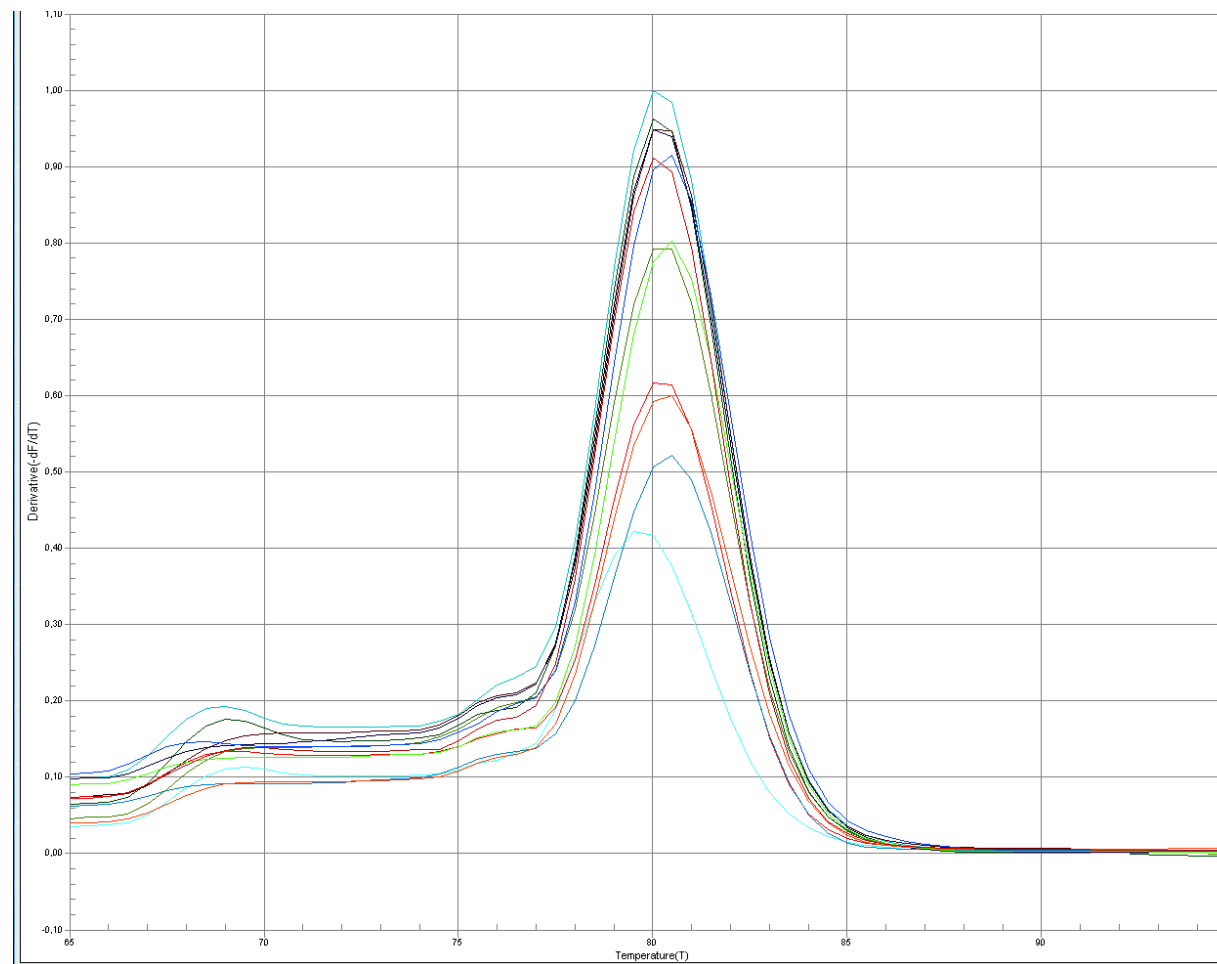

***EF1- $\alpha$***

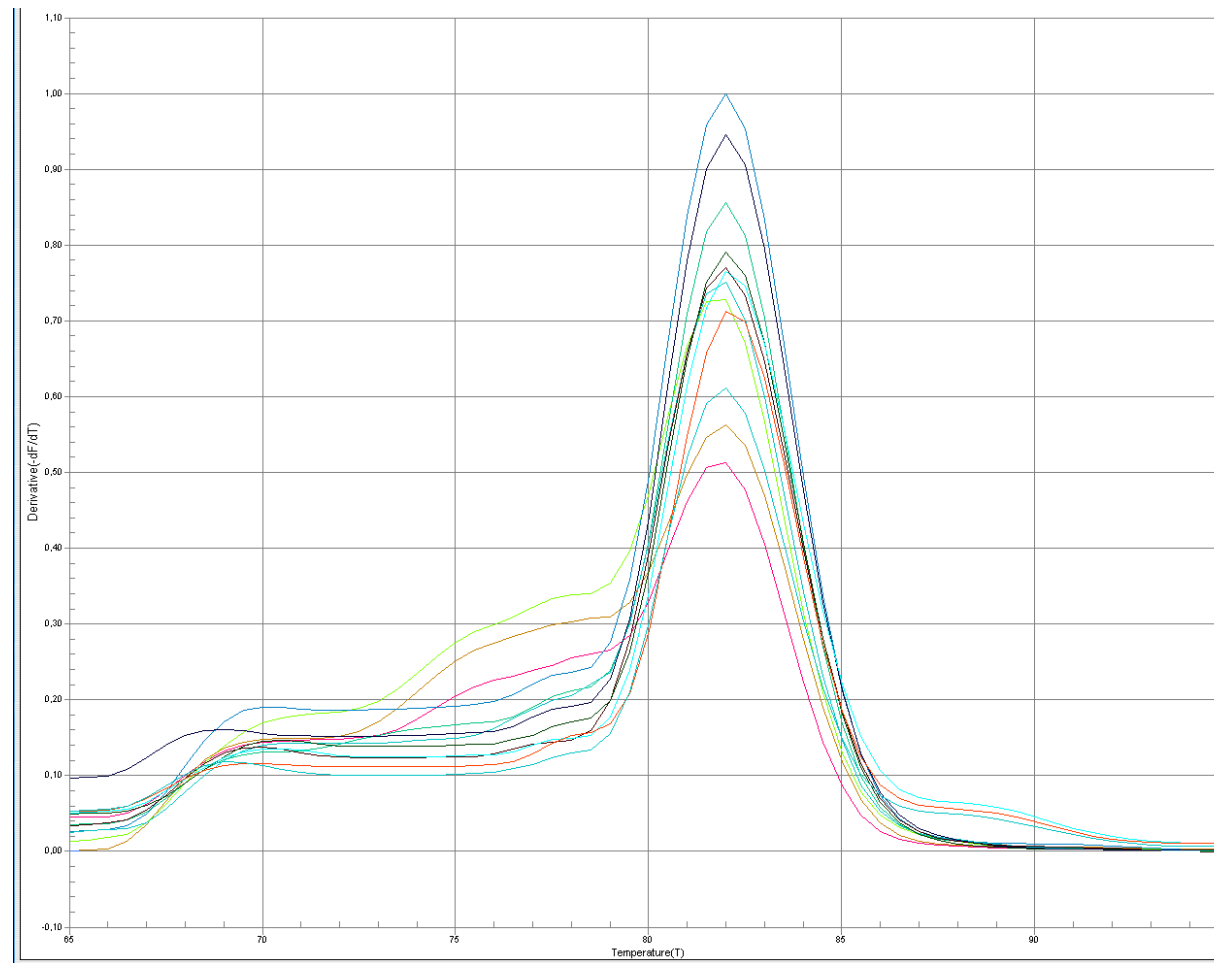

***UE21D***

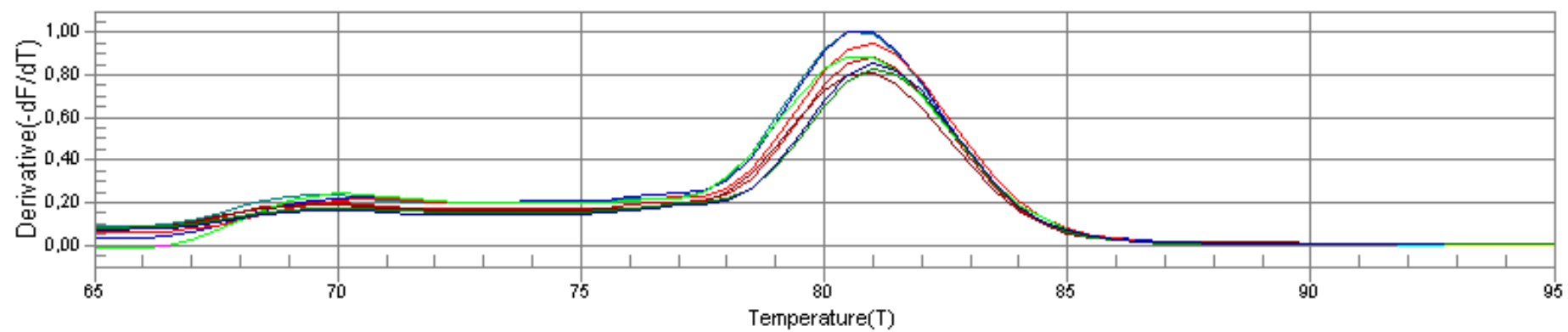

ZMP

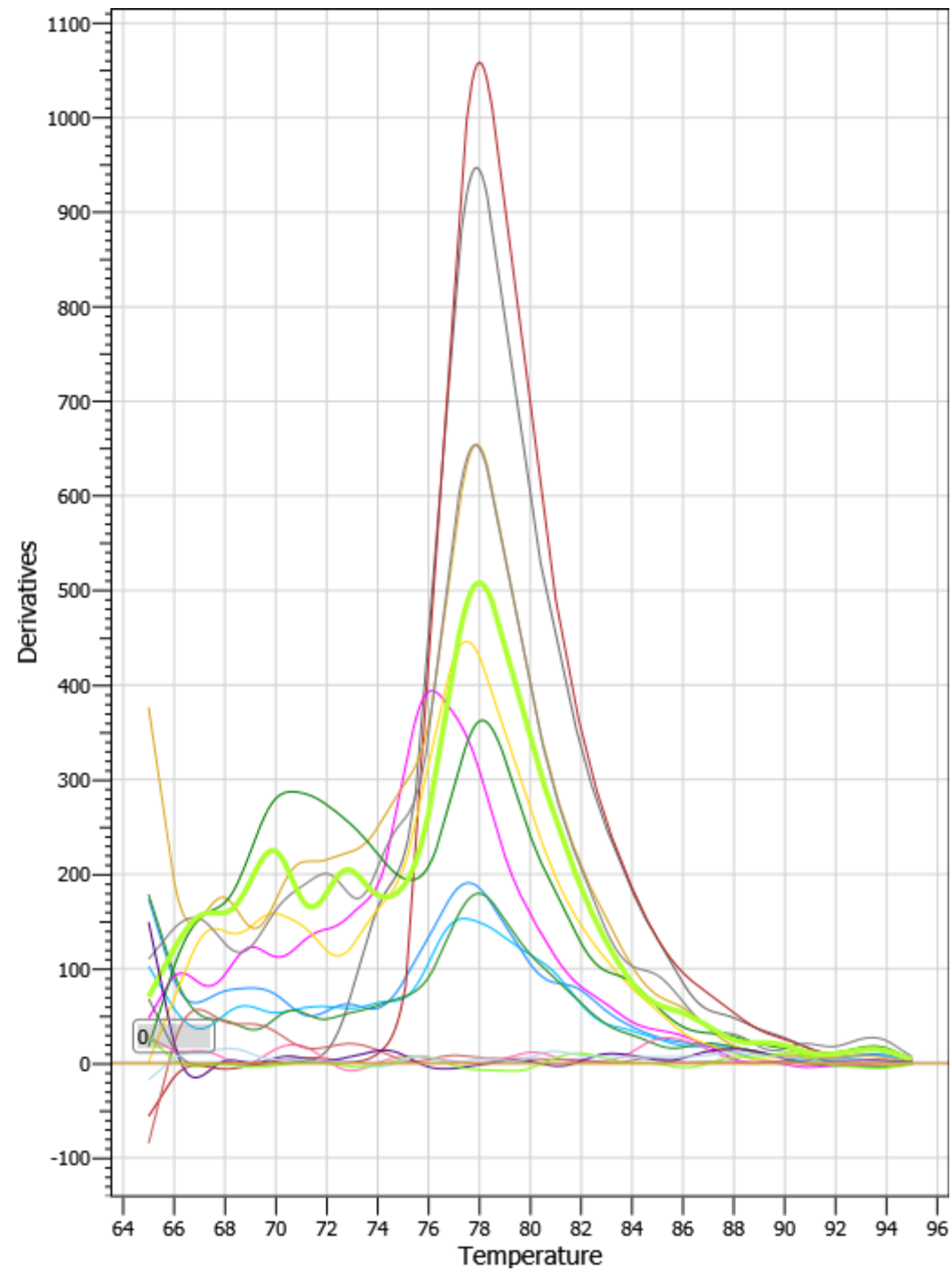

***GAPC***

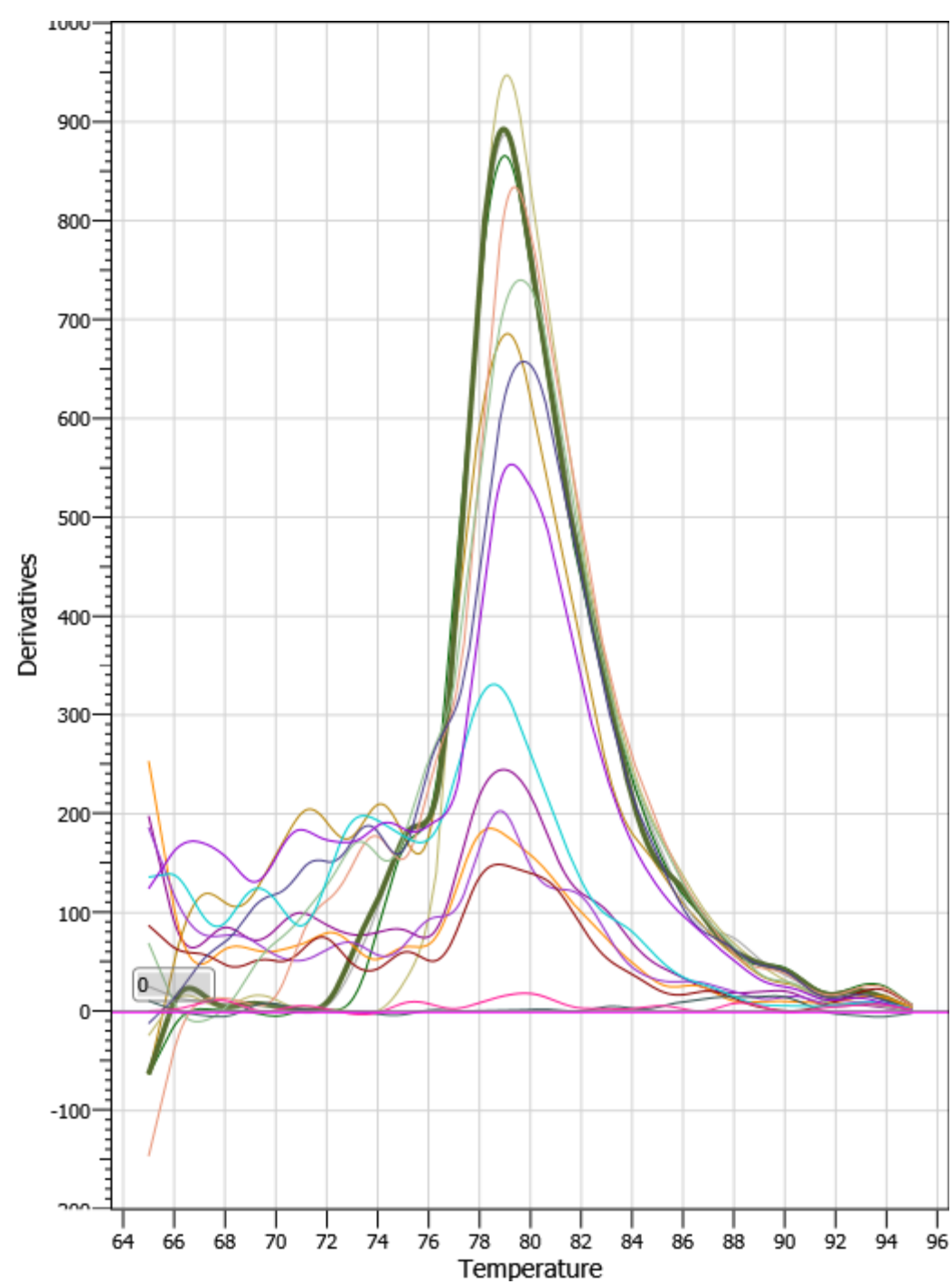

Supplement: Supplementary file 5 — Additional file 5. Appendix S2. Melting curve for seven candidate reference genes [(FBOX) F-box protein; (VuACT) actin; (VuUBQ10) polyubiquitin 10; (eEF-1α) eukaryotic elongation factor 1α; (β-TUB) beta-tubulin; (UNK) Phaseolus vulgaris unknown gene; (UE21D) ubiquitin-conjugating enzyme E2 variant 1D] with single peak. [file 13007_2018_354_MOESM5_ESM.pdf]
