# Supplementary material for: Cowpea and abiotic stresses: identification of reference genes for transcriptional profiling by qPCR
Source: Plant Methods. 2018 Oct 12;14:88. doi: 10.1186/s13007-018-0354-z (PMC6182843; doi:10.1186/s13007-018-0354-z)
Supplement: Supplementary file 2 — Additional file 2. Appendix S1. Sequences used in the present work. [file 13007_2018_354_MOESM2_ESM.pdf]

Supplementary appendix 1. Sequences used in the present work.

Arabidopsis (Ubiquitin10) and *Vigna radiata* (actin) queries

Arabidopsis thaliana polyubiquitin 10

NCBI Reference Sequence: NM_001084884.4

>gi|240255763|ref|NM_001084884.4| Arabidopsis thaliana polyubiquitin 10 mRNA, complete cds
TCACAATTCAGATTTCAATTTCTCAAAATCTTAAAAACTTTCTCTCAATTCTCTCTACCGTGA
TCAAGATGCAGATCTTTGTTAAGACTCTCACCGGAAAGACAATCACCCTCGAGGTGGAAAGC
TCCGACACCATCGACAACGTTAAGGCCAAGATCCAGGATAAGGAGGGCATTCCTCCGGATC
AGCAGAGGCTTATTTTCGCCGGCAAGCAGCTAGAGGATGGCCGTACGTTGGCTGATTACAAT
ATCCAGAAGGAATCCACCCTCCACTTGGTCCTCAGGCTCCGTGGTGGTATGCAGATTTTCGTT
AAGACGTTGACTGGGAAAACTATCACTTTGGAGGTGGAGAGTTCTGACACCATTGATAACGT
GAAAGCCAAGATCCAAGACAAAGAGGGTATTCCTCCGGACCAGCAGAGATTGATCTTCGCC
GGAAAACAACTTGAAGATGGCAGAACTTTGGCCGACTACAACATTCAGAAGGAGTCCACAC
TCCACTTGGTCTTGCGTCTGCGTGGAGGTATGCAGATCTTCGTGAAGACTCTCACCGGAAAG
ACCATCACTTTGGAGGTGGAGAGTTCTGACACCATTGATAACGTGAAAGCCAAGATCCAGG
ACAAAGAGGGTATCCCACCGGACCAGCAGAGATTGATCTTCGCCGGAAAGCAACTTGAAGA
TGGAAGAACTTTGGCTGACTACAACATTCAGAAGGAGTCCACACTTCACTTGGTCTTGCGTC
TGCGTGGAGGTATGCAGATCTTCGTGAAGACTCTCACCGGAAAGACTATCACTTTGGAGGTA
GAGAGCTCTGACACCATTGACAACGTGAAGGCCAAGATCCAGGATAAGGAAGGAATCCCTC
CGGACCAGCAGAGGTTGATCTTTGCCGGAAAACAATTGGAGGATGGTCGTACTTTGGCGGAT
TACAACATCCAGAAGGAGTCGACCCTTCACTTGGTGTTGCGTCTGCGTGGAGGTATGCAGAT
CTTCGTCAAGACTTTGACCGGAAAGACCATCACCCTTGAAGTGGAAAGCTCCGACACCATTG
ACAACGTCAAGGCCAAGATCCAGGACAAGGAAGGTATTCCTCCGGACCAGCAGCGTCTCAT
CTTCGCTGGAAAGCAGCTTGAGGATGGACGTACTTTGGCCGACTACAACATCCAGAAGGAG
TCTACTCTTCACTTGGTCCTGCGTCTTCGTGGTGGTTTCTAAATCTCGTCTCTGTTATGCTTAA
GAAGTTCAATGTTTCGTTTCATGTAAAACTTTGG

Vigna radiata actin

NCBI Reference Sequence: XM_014655942.1

>XM_014655942.1 PREDICTED: Vigna radiata var. radiata actin-1-like (LOC106770112), mRNA
CCCGTTGTTTTTCTCCGTAAAAGAATGAATAGTATTGGTGTAAACGGAGAAAAGAATGAGCG
ATAAATGTAGCAGTAGCAGCGTGAATTTCTCTTTCTCTTTCTCCAAATTTAAAATGGACAAA
AATTGGGAGGCATGATCCGACGGTCCCGATTGGGTTTGATGGTTGTTATATTCATTTGAATG
GAATTTCGTTCATTTTGTGTTTGTCACTGAGAATCGATTTTTCATCCTCTCTTCTCCTCTTCTCT
CCTCTCTCTCGCACTCGCATTTCCAAAGCAGAGCAGATATTAGAAGATGGCAGACGCTGAGG
ATATTCAACCCCTTGTTTGTGATAATGGAACGGGAATGGTTAAGGCGGGGTTTGCGGGAGAT
GATGCCCCACGTGCTGTCTTCCCTAGCATTGTTGGCCGTCCTCGCCACACTGGTGTGATGGTT
GGCATGGGCCAGAAAGATGCGTATGTGGGTGACGAGGCTCAGTCTAAGCGAGGTATTCTCA
CTCTCAAGTACCCCATTGAGCATGGTATCGTCAGCAACTGGGATGATATGGAAAAGATTTG
GCATCATACCTTCTACAATGAGCTTCGTGTCGCCCCAGAGGAACATCCCGTTCTCTTGACCG
AGGCTCCTCTTAACCCTAAGGCTAATCGCGAGAAGATGACCCAAATCATGTTTGAGACTTTT
AATGCCCCTGCCATGTATGTAGCCATTCAGGCTGTTCTTTCACTCTATGCCAGTGGTCGTACA
ACCGGTATTGTGTTGGATTCTGGGGATGGTGTCAGTCACACAGTCCCTATCTACGAGGGTTA
TGCCCTTCCTCATGCCATCCTTCGTCTTGATTTGGCTGGTCGTGATCTCACTGATTTTCTGATG
AAGATTCTGACTGAACGTGGATATTCTTTTACCACCTCAGCGGAGCGCGAAATTGTGAGGGA
TATGAAGGAAAAGCTGGCTTACATAGCCCTGGACTATGAGCAGGAGCTTGAGACAGCCAAG
ACCAGCTCTGCAGTGGAGAAGAGCTATGAGTTGCCTGATGGGCAGGTTATCACCATTGGCGC
TGAGCGTTTCAGGTGTCCCGAGGTCTTGTACCAACCATCCATGGTAGGAATGGAAGCAGCAG
GCATTCACGAAACAACATACAACTCCATTATGAAATGTGATGTTGACATCAGGAAAGATCTG
TACGGTAACATTGTACTTTCAGGAGGCACAACCATGTTCCCTGGCATTGCTGATAGAATGAG
CAAGGAAATTTCTGCTTTGGCCCCCAGCAGCATGAAGATCAAGGTGGTTGCACCTCCTGAGA
GAAAGTACAGTGTCTGGATCGGAGGTTCTATCCTGGCATCCCTGAGCACCTTCCAGCAGATG

TGGATTGCAAAGGCAGAGTACGATGAGTCTGGACCATCAATTGTGCATAGAAAGTGCTTCTA
AATTGTTTGATCAATAGGGTTATTGAAGGGAAAGACGGCTGTTTTATTCCTCCGGAAGAAGT
GCTGCCTGCTTGTAACCCTTTCATTCTATTCTAACTTTTTGTATCCGTTTACGTTATTCTCTGG
AGTGATGATTTGGAGAAGGCGAGGAGTTTATTTTGTCAAAAATGGTGCATGCAATTTTCACT
ATTATTATATATTTCTATTTCTATTTTGTTTAGATTCATTGTGCGTCTGAATCAGCTGAAACTA
TCAAATGAACCACGCATTCTTGTTCTTGATATACAAAATTATGATTCCGATTTTCTGCATTA

Anchors sequences of CRGs primers pairs (fragments highlighted in red indicate the anchoring sites of the primer pairs)

1. **CRG**: *Vigna unguiculata* Actin

Database: NordEST

Sequence:

>Contig16004

GCGCTCTTTCATCCTCTCTTCTCCTCTTCTCTCCTCTCTCTCGCACTCGCATTTCCAAAGAGCA
GATATTAGAAGATGGCAGACGCTGAGGATATTCAACCCCTTGTTTGTGATAATGGAACAGGA
ATGGTTAAGGCGGGGTTTGCGGGAGATGATGCCCCACGTGCTGTCTTCCCTAGCATTGTTGG
CCGTCCACGGCACACCGGTGTGATGGTTGGCATGGGCCAGAAAGATGCGTATGTGGGTGAC
GAGGCTCAGTCCAAGCGTGGTATTCTGACTCTCAAGTACCCCATTGAGCATGGCATCGTCAG
CAACTGGGATGATATGGAAAAGATTTGGCATCATACCTTCTATAACGAGCTTCGTGTCGCCC
CTGAGGAACATCCCGTTCTCTTGACCGAGGCTCCTCTTAACCCTAAGGCTAATCGCGAGAAG
ATGACCCAAATCATGTTTGAGACTTTTAATGCCCCTGCCATGTATGTAGCCATTCAGGCTGTT
CTTTCACTCTATGCCAGTGGTCGTACAACCGGTATTGTGTTGGATTCTGGGGATGGTGTCAGT
CACACAGTCCCTATCTACGAGGGTTATGCCCTTCCTCATGCGATCCTTCGTCTTGATTTGGCT
GGTCGTGATCTCACTGATTTTCTGATGAAGATTCTGACTGAGCGTGGATACTCTTTTACCACC
TCAGCAGAGCGTGAAATTGTGAGGGATATGAAGGAGAAGCTGGCATACATAGCCCTGGACT
ATGAGCAGGAGCTAGAGACATCGAAGACCAGCTCTGCAGTGGAGAAGAGCTACGAGTTGCC
TGATGGGCAGGTTATCACCATTGGAGCTGAGCGTTTCAGGTGTCCAGAGGTGTTGTACCAAC
CATCCATGGTGGGGATGGAAGCAGCAGGCATTCACGAAACAACATACAACTCCATTATGAA
ATGTGATGTTGACATCAGGAAAGATCTGTACGGTAACATTGTACTTTCAGGAGGCACAACCA
TGTTCCCTGGCATTGCTGATAGAATGAGCAAGGAAATTTCTGCTTTGGCCCCCAGCAGCATG
AAGATCAAGGTGGTTGCTCCTCCTGAGAGAAAGTACAGTGTCTGGATCGGAGGTTCTATCCT
GGCATCCCTCAGCACCTTCCAGCAGATGTGGATTGCAAAGGCAGAGTACGATGAGTCTGGA
CCATCAATTGTGCATAGAAAGTGCTTCTAAATTGTTTGATCAATAGGGTTATTGAAGGAAAA
GACGGCTGTTTTATTCCTCCGGAACAACTGCTGCCTGCTTGTAACCCTTTCATTCTATTCTAA
CTTTTTGTATCCGTTTACGTTTTTCTCTGGAGTCATTCATGATTTGGAGAAGGCGAGGACTTT
GTTTTGTCAAAAATGGTGCATTCAATTTTCCCTATTATTATATATTTCTATTTTTATTTTGAAT
TGAGATC

---

2. **CRG**: *Vigna unguiculata* polyubiquitin 10

Database: NordEST

Sequence:

>Contig282

AGTGGGGACAGTTCAATTCAAAACAAACTACTTCATTCTCTTTGCGCAGTTCCCTACCTCTCC
CCTCAAGATGCAGATCTTCGTTAAGACCCTTACCGGAAAGACCATCACTCTCGAGGTCGAGA
GCTCCGACACTATTGACAACGTGAAGGCCAAGATCCAGGATAAGGAAGGAATTCCCCCAGA
CCAGCAACGTCTCATCTTCGCTGGAAAGCAGTTGGAGGATGGACGCACCCTCGCCGACTACA
ACATCCAGAAGGAATCAACCCTTCACCTAGTTCTCCGTCTCCGCGGTGGCATGCAGATCTTT
GTGAAGACTCTCACTGGCAAGACCATCACTCTTGAGGTCGAGAGCTCCGACACAATTGACAA
CGTGAAGGCCAAGATCCAGGATAAGGAAGGAATTCCACCAGACCAGCAACGTCTCATCTTC
GCTGGAAAGCAGCTGGAGGATGGTCGCACCCTCGCCGACTACAACATCCAGAAGGAATCAA
CCCTTCACTTAGTTCTCCGCCTCCGCGGTGGCATGCAGATCTTTGTTAAGACTCTCACCGGCA
AGACCATCACCCTTGAGGTTGAGAGCTCTGACACCATCGACAACGTGAAGGCCAAGATCCA
GGACAAGGAGGGAATTCCCCCGGACCAGCAGAGGTTGATCTTCGCCGGAAAGCAGCTGGAG
GATGGAAGGACCCTGGCCGACTATAACATCCAGAAGGAGTCAACTCTCCACTTGGTGCTGCG
TCTTCGTGGTGGTATGCAGATCTTTGTGAAGACCCTCACTGGGAAGACTATTACCCTTGAGG
TGGAGAGTTCGGACACTATTGACAATGTGAAGGCCAAGATCCAGGACAAGGAGGGTATTCC
CCCGGACCAGCAGAGGTTGATCTTCGCTGGGAAGCAGCTTGAGGATGGGAGGACCCTTGCT
GACTATAACATTCAGAAGGAGTCTACCCTTCACCTTGTCCTTCGTCTAAGGGGAGGAATGCA
GATTTTCGTTAAGACTCTTACGGGCAAGACCATTACCCTTGAGGTGGAGAGCTCTGACACCA
TTGACAACGTGAAGGCCAAAATTCAGGACAAGGAGGGCATCCCACCGGACCAGCAGAGGTT
GATCTTTGCAGGGAAGCAGTTGGAGGATGGAAGGACCCTTGCTGATTACAACATTCAGAAG
GAGTCAACTCTTCACCTTGTCCTCCGTCTTCGTGGGGGCTTTTAGACTGTGCTGATCTCTGTTT
TTAGTGCTTTCAAGACATTTTCAAAATCGTAATGTGGATTGGGTCTTTATTGACCCTTTAAAT
AAATTTGGTTTGTGTTGGACTGCATTGTCCCTTTTTTATTATGAAACTTTAGATATATACTATG
CTATTCTATGTTGCCTTATGTATTGATTTACGATTATTAATCAAGTTTAGATATATATATATAT
AATAACTAATTATGCTATT

3. **CRG**: Beta-tubulina

Database: RefSeq (NCBI)

Sequence:

>XM_007147394.1 *Phaseolus vulgaris* hypothetical protein (PHAVU_006G126100g) mRNA, complete cds

TAGCATTCACCCCTCTCATTCATTTCCATTTCCTTCAAACCCAATCCCCAGTTTCCTCAAACCC
TAATTCCCTTTCTCCCACACTCTTGCACACTCTCCGATCGAAAATGAGAGAAATCTTGCACAT
CCAGGGAGGGCAATGCGGGAACCAGATCGGAGCCAAGTTCTGGGAGGTGATCTGCGACGAG
CACGGCATCGACCACACCGGGAAGTACAGCGGCGATTCCGAGCTCCAACTCGAACGCATCA
ACGTCTACTATAATGAAGCCAGCGGCGGAAGGTACGTTCCACGCGCCGTCCTCATGGATCTT
GAACCCGGCACCATGGACTCCGTCAGATCCGGCCCCTACGGCCAGATCTTCCGCCCCGACAA
TTTCGTCTTTGGCCAGTCCGGCGCCGGCAACAACTGGGCCAAAGGCCACTACACTGAAGGTG
CCGAACTCATCGACTCAGTCCTCGACGTCGTTCGCAAAGAAGCCGAAAATTGCGATTGCTTG
CAAGGGTTTCAGGTGTGCCATTCTCTTGGGGGAGGAACGGGTTCTGGCATGGGGACGCTTCT
GATTTCAAAGATTCGTGAGGAGTATCCAGATCGGATGATGTTGACGTTTTCAGTGTTTCCTTC
TCCTAAGGTTTCTGACACCGTTGTGGAGCCTTACAATGCTACGCTCTCTGTTCACCAGCTTGT
AGAGAACGCTGATGAGTGTATGGTTCTGGACAATGAGGCTCTCTACGACATTTGTTTCAGGA
CCCTCAAGCTCGCTACACCGACGTTTGGTGACCTTAACCACCTGATTTCCGCCACCATGAGT
GGAGTTACTTGCTGTCTACGTTTCCCTGGGCAACTGAACTCCGATCTTCGCAAGCTTGCTGTT
AATCTTATCCCATTTCCCCGGCTCCATTTCTTTATGGTTGGGTTTGCGCCCTTGACATCTAGA
GGATCCCAGCAGTACCGTGCTTTGACTGTTCCTGAATTAACACAACAAATGTGGGATGCTAA
GAACATGATGTGTGCTGCTGATCCTCGTCACGGTCGTTATTTGACTGCATCAGCAATGTTCCG
TGGTAAGATGAGCACAAAGGAGGTAGATGAGCAAATGATCAATGTGCAAAACAAGAACTCT
TCATATTTCGTTGAGTGGATACCTAATAATGTGAAGTCCAGCGTGTGTGATATCCCGCCTAA
GGGTCTTAAAATGGCTTCTACTTTCATTGGCAATTCTACTTCAATCCAGGAGATGTTCAGGAG
AGTTAGTGAGCAGTTCACGGCTATGTTCAGGCGCAAGGCTTTCTTGCATTGGTACACTGGGG
AAGGAATGGACGAAATGGAGTTCACTGAGGCTGAGAGTAACATGAATGATCTTGTTGCCGA
GTATCAGCAGTACCAGGATGCTACCGCCGATGAGGATGAGTACGAGGAGGAAGAAGAAGAT
GAGGAGGAAATTGCACCATAAGGCACTCCTTCATTTATAAAATGGTTTCAAATATCAATGTG
GTTGGCTTTGAACGAGTGGTGTAAGTTTTAATTGTGATGCAGTCAAATTAGATTGACTCGTTA
ATGTGTATGCTTATTATTGATTGTTGACATTGTATAAACTGGGTTTTGTTGACAATATTTCTGT
TCTTGAGAGATCAAGTCTTGGATGTTTGTAATAGTTAGTGACATGTTTTTTTCTCTTC

Annotation in Phytozome:

Transcript Name: Phvul.006G126100.1 (primary)

Description: PTHR11588:SF97 - TUBULIN BETA-4 CHAIN-RELATED

---

4. CRG: Elongation factor 1-α

Database: RefSeq (NCBI)

Sequence:

>XM_007151727.1 *Phaseolus vulgaris* hypothetical protein (PHAVU_004G075100g) mRNA, complete cds

CGCACAGCAAGCGAAAGCAAAAAGGATCCAAATGGAACTACTTAAAATAATAAGGGTATTT
TCGTCAACAGAGGAAAAAACTTGTAGCCTATAAATAAACCCTCTAACCCTCGTTTCCTCACT
TCTCTTCACTCTATCATTCTCCTTTCTTCTCTTGCGGCTAGGGTTTTAGCGCAGCTTCTTCTAG
ATACTCAAACGCAGTTATGGGTAAGGAAAAGGTTCACATCAGTATTGTGGTCATTGGTCATG
TCGACTCTGGGAAGTCCACCACCACTGGCCATCTGATCTACAAGCTTGGAGGCATTGACAAG
CGTGTGATTGAGAGGTTCGAAAAGGAAGCTGCTGAGATGAACAAGAGGTCATTCAAGTATG
CCTGGGTGCTTGACAAGCTCAAGGCTGAACGTGAAAGAGGAATTACAATTGATATTGCCTTG
TGGAAGTTTGAAACCACTAAGTACTACTGCACAGTCATTGATGCTCCCGGACACAGGGATTT
CATTAAGAATATGATTACCGGAACATCTCAAGCTGACTGTGCTGTTCTCATCATTGATTCTAC
CACTGGTGGTTTTGAAGCTGGTATTTCTAAGGATGGACAGACTCGTGAACATGCTCTTCTGTC
TTTCACTCTTGGTGTGAAGCAGATGATTTGTTGCTGTAACAAGATGGATGCTACTACACCCA
AGTACTCCAAGGCTAGGTATGATGAAATTGTGAAGGAAGTCTCTTCCTACTTGAAGAAGGTG
GGATACAACCCTGACAAAATTCCTTTCGTACCAATTTCTGGTTTTGAGGGAGACAACATGAT
TGAGAGGTCCACAAACCTCGACTGGTACAAGGGACCTACCTTGCTTGATGCACTTGACCAGA
TCTCTGAGCCCAAGAGGCCATCTGACAAGCCTCTCAGGCTTCCTCTTCAGGATGTTTACAAG
ATTGGAGGAATTGGAACTGTGCCTGTGGGACGTGTTGAGACTGGTGTCATCAAGCCTGGTAT
GGTGGTGACTTTTGCACCATCTGGACTGACAACTGAAGTTAAGTCTGTGGAGATGCATCATG
AAGCTCTCACAGAGGCTCTCCCCGGTGACAACGTGGGGTTCAATGTGAAGAATGTTGCTGTT
AAGGATCTCAAGCGTGGTTATGTTGCTTCAAACTCCAAGGATGATCCTGCCAAGGAGGCTGC
TAACTTCACCTCCCAGGTTATCATCATGAACCACCCTGGTCAGATTGGAAATGGCTATGCCC
CTGTTCTGGATTGCCACACCTCCCACATTGCTGTCAAGTTTGCTGAACTTATGACCAAGATTG
ACAGGCGATCTGGCAAAGAGCTCGAGAAGGAGCCTAAGTTCTTGAAGAATGGTGATGCTGG
ATTTGTTAAGATGATTCCAACCAAACCCATGGTGGTGGAGACTTTCTCTGAGTATCCCCCTCT
TGGTCGTTTTGCTGTGAGGGACATGCGTCAAACTGTTGCTGTGGGAGTCATCAAGAGCGTGG
AGAAGAAGGATCCCACTGGAGCCAAGGTTACCAAGGCCGCACAGAAGAAGAAGTGAATCG
TGCTGGGCGGTTCAACAGGGGATGGTTATAATAAATGCTGGTTTCTTGCTTGTACTCTTGTGT
CATCGCTAGGTAGATTGTTTTTCGTACTTAGTTTGAAGTTTCCGCCATCATCTCCGAACTTTT
GTTCCCAGAATTGGGTTCTTGATCGACGGTGGCGGCTCTTTTACTATTATTATGTCTTTTTATG
TCTTTTTGTGTTTTTGTGAGAACCCCTGATTACATTTTTGTAAGCGCAGCAAGTTTTACGGCTT
TGCTGCGTGGTTTGTTTGCATTTTAAATATCGGACTTTTATATTTGTGTTTTAAATCGTGTTTC
CTAATTATTTTCAGCATTTTAAATGTTATCTGTCATTTCTGTCGTTTTAAATATTATCTGCCTT
GCATTAT

Annotation in Phytozome:

Transcript Name: Phvul.004G075100.1

Description: K03231 - elongation factor 1-alpha (EEF1A)

5. **CRG**: F-Box protein

Database: RefSeq (NCBI)

Sequence:

>XM_007131876.1 *Phaseolus vulgaris* hypothetical protein (PHAVU_011G053400g) mRNA, complete cds

GAATAGTTTGTTCTCTGTCAAATAATTTTGCTTTAAATGTTATAGTTATGAACAAATTTGGTT
GATTGTTGAGAGAGTGCAATTGAGAGAGCAGAAAAATGGGGTTGGAATCTGTTGGAGATTT
AGCCATTAACATGATTCTTAAGAAGTTAGAAGGCGAAGATGTTGCCAGAGTCGCTTGTGCCA
GCAAAAGGTTTAGGTTTTCAGCTTCAGATGACACTCTTTGGACCAATCTCTGCTTCCATGAAC
TCGCTTTGACCCAACCCATCGATCATCTTGGAAACCCCTTCCCTTCCTTCAAGGAATGCTATC
AAGCATGGAGAGGAGCTTTTGATATGTATCCATGGCCTCTTGTTAAGCGTGTGAAAAAGTGC
TGGGATAGAATAAAGACCTGGTTGACCAATAACTTTCCTGAAGCTGAGGCCACTCTTTGTAA
AGGGGCAACTGAAGCTGACATTCAGACGTTGGAGAATTTGTTAAAGGTGGAATTGCCACTTC
CTACAAGGATCCTTTATCGCTTTCATAATGGGCAAGAAATCGTAAAAGCAAATCCAGAAACT
AGTACATTTGGAAGTTCTTTGGGTCTAATTGGGGGTTACTCCTTCTATAATCATTTGGTGAAT
GTTTATCTATTGCCTATACGTCAGATAATCCAAGAAACTGAGCAAATTAGGCGTCACTTGAG
CTTTTTAAGAAGATCCAAGTTTGTTCTTGTGGCTGCTTCATCCACTTTCCGTAGAAAGTTGTT
TTTCCTCAACTGTTCCAATGGTCAACTATATGTTGGAACCAATAAACTTCATTCTGAAAAAG
ACATAATCCCTTGTGTACCTCAAGATCTGATAAGTTTACATCGGGAATTAAATATTGAAGAG
CAACAAGATGCCATGCTACTGTGGTTAGAAGAACATGGTCGTCGTTTAGAACATGGCTTCAT
CAAACTCTGTGACAAAGAAAATGGAAGAAGCATTAATCTTTTCCCAGAAGAACCCCCTTTTT
GTTCAACGGCTGTTACTAATGGTGTGAAGGTCCGTGCATCTGCATTGATTATCCCTGAGTTTA
TTGATCCTCAAGATGAGAATGAAAAGTACTTATTTGCTTATTCAATCCGCTTGTCCCTTGAAC
CGCAAGGATGCTTGATTAATGGAATGTCCTTCAACTCTTGTCAGCTCCATTGGAGGCACTGG
ATCATTCGTGCTAATGATGATGTTGTATCGGATTTCAATGGAGAAGCTGTTATAGGACAGTA
TCCACTTCTGCATCCTGGTGCAGAAGAATTTGTTTATCAGAGTTGCACACCTCTACCACTGCC
ATCAGGTTCCATTGAAGGTTCTTTTACTTTTGTACCTGGCAGCTTGGCATACCCAAAAGGAG
ACCCTTTTCTAGCTACAGTGGCACAATTCCCTCTCCAGCTGCCAGACTATATTTTCTGATTTT
GATTCTGAATGGAAATGAAGTATCTCTGATGGCAATTGCAGCTCCCCAATGGCGTTTGTGCA
CATTATGTGACTTTAGTGAGAGTGGCTTGGTTCCTGAGTTTTTATTCTTCTAAGTGTTGGACC
TGCACAATTTCCCTATATATAATCTATCTGTAGTAATATTGGATTTCTTGCGTTATTCACTTGT
CTCTTAACTTTGTACCCTCCATTCACTCTGTATATATGCATGCGATTCAATGAGGTTTTTGTCA
TCTAATAGAATGGTAAAAG

Annotation in Phytozome:

Transcript Name: Phvul.011G053400.2

Description: PTHR14289:SF16 - F-BOX ONLY PROTEIN 3

6. **CRG**: Ubiquitin-conjugating enzyme E2 variant 1D

Database: RefSeq (NCBI)

Sequence:

>XM_007145751.1 *Phaseolus vulgaris* hypothetical protein (PHAVU_007G270100g) mRNA, complete cds

AAAAATTGGGCCGAAACAGAACGGGCCCTTTTGGTCCATAGATTAGCAGTGAGGGACCAAT
AGTCATTATTCATTGCGAAAATCTTTCATCATTCTTTCTTTCTACAAAATCCCTCATTTCCTTC
TTTCTTCCCATTTCCCACCTTTTCATCATCGTTCATCGCCTCCACCACGCACTCACCCAGATTC
TCCTTTCTCTCTGCCTTCTTCTTCCCTCTCCAAATTTTGATTCTTTCGATCAGATCCGCCACCC
CCACCTCCCCAAAACCCTCTGCCACCCATCCATGACGCTTGGCTCAGGAGGATCCAGTGTCG
TGGTTCCAAGGAACTTCAGATTGCTGGAGGAGCTTGAACGAGGAGAAAAAGGTATTGGAGA
CGGCACAGTTAGCTATGGAATGGATGATGGTGATGACATCTACATGCGATCTTGGACTGGCA
CCATTATTGGCCCCCATAATACTGTACATGAAGGAAGAATCTATCAACTGAAGCTGTTTTGT
GATAAAGACTACCCAGAAAAAGCCCCCAAGTGTTCGGTTTCATTCACGGATCAACATGACCTG
TGTTAATCATGAAAATGGAGTGGTTGAACCGAAGAAGTTTGGTCCTCTTGCAAATTGGCAAA
GAGAGTACTCCATGGAGGATATACTGACCCAGCTGAAGAAGGAGATGGCAGCTCCTCATAA
CCGGAAGCTTGTGCAGCCCCCAGAAGGAACCTACTTTTAGCATTGAAGACGGTATAGATGTC
AGCTTGTATTGCATATGCTGTATGCAATATATAGTGTGTAATGCTTTGTGGATCCTTCCTTCT
GAATTCAAGAACATATAGGGGAATGCCCCAAAAGATATTTCCATCTCCTTGCCTTCAAAGTT
GTTTTGTTGTCTTTCTCAACTGTGCTGAAGGGGGACTGTTTGATTAAAATATTGTGTGTAAAA
TATTTCTAAAGCAACTATTTTAAGTAGTATTTTCCCTTTAATCCCAAAGGTGCTTTCAATTTTA
GTCTTGCT

Annotation in Phytozome:

Transcript Name Phvul.007G270100.1

Description:PTHR24067:SF117 - UBIQUITIN-CONJUGATING ENZYME E2 VARIANT 1D

7. **CRG**: Unknown1

Database: RefSeq (NCBI)

Sequence:

>XM_007131494.1 *Phaseolus vulgaris* hypothetical protein (PHAVU_011G023200g) mRNA, complete cds
GTGAAGAGCGAGGTGTGCATCGCAAATTCTAATTTTCTGATAAAACTAATAATAATAACAAC
AAAACAGACACACCTGTGTAAGAAGGAGGTGAGTTACGTACGATTAGGTTTTCTTCTTCTCT
TTCTCTCTGATTCGATCCATTTCGATTGCTGAATTCATCCCATCGCAGAGCGTTTGAATTTGT
AGTTGAACAACGATGGTGGTGCCGCTGCTTAGTGTTTTCATTCTTTTCGCTTTTATTCCAAAC
GGATTGTTAGCAGTTCCTTCAACCGTCCCCGCATTCCTTTGGTCATCACATTATGAACTGGCC
TCTGAAACTGGATTGAAGGAATCTGTAAATTACCAAGTCATTTCTCCTTATGATCTTGCAAA
GTCTGTTATATCTGAAGCGGGCTGGTCAAATTTTCTGTGCAAAGGAAAGAAAACTCACAAGC
CTCTGGATCTGGCACTTTTGTTCGTTGGTGGAGAGCTGCAATCTTCAGATTTAAGCATGAACA
AACATGCAGACCCAGCCCTTTTAGATCTGCTCAAGATCTCTTTTGCCAGATCCAACACTTCTA
TGGCTTTTCCCTATGTTTCTGCATCAGAGGATTTGCTTTTGGAAAACACTTTGGTTTCAGGAT
TTTCTGAAGCCTGTGGAGATGATACGGCAATTGCCAATGTTGCGTTCCATGGATCTTGCTCCA
TGGGTGGTACAAATGAAGAAGTCACAGCTTTGCATTCAGTACAAGACTATTTGACAAAGAG
GATGAAAGATAGTCACAGGGGGAAAACAGATTTGGTTGTGTTCTGCAATGGAGGCTCTCAA
GATCTTAAAAATGTTGACAGGACACAATCTGAAGGGGAAACTTTATCGAAGCTTCTGAGTTC
CATGGAGGAATCGGGTGCAAAATATGCTGTTCTTTATGTGTCAGATCCCTCCAGGTCAATCC
GGTATCCTTCTTACAGAGAACTGCAAAGGTTTCTTGAAGAAGGTGCTGAATCAACCAATTCC
ACAGGTTGTGATAAAGTCTGCATGCTTAAATCATCTCTTTTGGAGGGAATTTTTGTGGGTTTA
GTTTTGCTAATCATTTTGATATCGGGCCTTTGCTGCATGATGGGAATTGACACCCCAACAAG
ATTTGAGACGCCACAAGAGTAATGATCCGTTTCTCGAAGCTAAAATTTGTTGTGATGCTCGG
TCTAATGATTAAAATATTGCTGTCGTATTATTTGCGATTCTTCCGGAGGCTCTGATAAGTATT
CTGTAGTTGATGACTTATCTCAGGGACTTTTAATATTGAATATTAAAATAATTTGTATCAGTA
TATAAAAATAAAACTTCAATGTTAGGTTTATTTTTGATCCCGCTACCACTAGTGTAAAATTTG
AAGAAATTTCAGATTGTGATGTATTATTGTTATTACTGTCTGCAAATTTGATCCATTCGTTTT
GCATAACTGCTGATCGATGCAGTTCCAAGT

Annotation in Phytozome:

Transcript Name:Phvul.011G023200.1

Description: FAMILY NOT NAMED

8. **CRG**: Zinc-metallopeptidase, peroxisomal-like

Database: RefSeq (NCBI)

Sequence

>XM_007162147.1 *Phaseolus vulgaris* hypothetical protein (PHAVU_001G1332000g) mRNA, partial cds

CAAATAGACTGAACCCATTACCAGTACAAAAACACGAGTATCGCAATGGACTCTGTTCTGTA
GCTAACACTCCACAGTCCACAAAATAGCGAAATAAGAGTTAGGAAAAGTAGAACAGAACAA
AACAAAACAGAGTCGTCGTCGTCTTTCCTTACTTTTACTCGTCACCGCGAAATATCAATCTTC
TGCAGCACTCATCTCAATTCGAGTTTCAAGCGCAAGGAACAGAACAGAACACGCAATCATT
ACCTTCCAACGCCACCAAGCCAGAACAAGAACGAGTTAGGGTTGTAATCAGAGCGAGGGAG
AAGATGGCAATTAAGGACGACGTGGAAATTGTGAAGGCTCGCATCGACAAGAGGGATTACA
GAAGAGTCGTGCTTCGCAACTCGCTCCAAGTGCTCCTCATTAGCGATCCCGTTACCGATAAG
TGTGCTGCTTCCATGAACGTTGGCGTCGGTTACTTCAGTGATCCTGCTGGCCTCGAAGGCCTC
GCCCATTTCCTCGAGCACATGCTGTTTTATGCGAGTGAAAAATACCCAGTTGAAGATAGCTA
CTCCAAGTATATTACTGAGCATGGAGGAAGCACCAATGCTTTTACAAGTTCTGAACAAACCA
ACTATCATTTTGAAGTTAACACAGACGGCTTTGAAGAGGCTCTGGACAGATTTGCTCAGTTC
TTCACTAAACCATTGATGTCTCCTGATGCCACCATGAGGGAAATTAAAGCTGTTGACTCTGA
AAACCAGAAGAATTTACTATCTGATGGATGGAGAATTAACCAGCTTCAGAAGCATCTGAGT
GACGAAGATCATCCATATCATAAGTTTAGCACAGGGAATTGGGACACTTTGGAAGTTAAGCC
TAAAGCAAAAGGATTAGACACAAGGAAGGAGCTTCTCAAGTTTTATGATGAAAACTATTCT
GCCAATTTAATGCATCTAGTTATATACACAAATGAAACCCTTGATAAAATTCAAAACCTTGT
AGAAGAAAAGTTCCAGGATATTAGAAACACCAGCAAAAGTTGTTTCCATCCTTGTGGTCAGC
CATGCAAATCAGAGCATTTGCAGATTCTTGTCAAAACTGTCCCAATAAAGCAAGGTCACAAA
TTGAGAATTGTATGGCCAGTAACCCCTGAAATTCATCATTACACTGAAGGGCCAAGCAGGTA
TCTTGGCCATCTCATTGGTCATGAAGGAGAAGGGTCTTTATATTACATCTTGAAAAAATTGG
GGTGGGCTACGGGCTTGTCTGCCGGCGAATCAGATTGGAGCTTGGAGTTTGCTTTCTTTACA
GTTGTAATTGATCTTACTGATTCTGGTCATGAGCACATAGAAGATATCATTGGGTTGTTGTTC
AAATACATTGAGCTTCTACAGCAGTCTGGTGTTTGCGAATGGATTTTTGAAGAGCTTTCTGCA
GTTTGTGAGACCAAGTTTCATTATCAGGACAAAATTCCTCCCGGTGATTATGTTGTTGATATT
GCATCAAATATGCAGTTTTATCCTGTGAAAGATTGGCTGACAGGATCATCCTTGCCTTTTAAG
TTTAGCCCAAATGTTATCCATATGGTACTAAATCAGCTTTCTCCGGACAATGTTCGAATTTTT
TGGGAATCTAAAAATTTTGAAGGGCTTACTGATAAGGTGGAGCCATGGTATGGAACTGCGTA
TTCCCTTGAAAAGATTACTGGCTCTGCTATTCAGGGGTGGATGGCTTCTTCTGCTGATGAAAA
TATGCATCTTCCAGCTCCTAACAAATTCATTCCAACGGACTTGTCACTTAAAACTGTGCAAG
AAACGGTGAAGTTTCCAGTTTTGTTAAGCAGGTCAACTTATTCAGCCTTATGGTACAAGCCA
GACACATTGTTTGCTACACCCAAGGCTTATGTTAAGATTGATTTCAATTGCCCATATGCTGGA
AGCTCTCCTGAGGCTCAGATTTTGGTGAATATTTTCACACAATTATTGATGGATTACTTGAAT
GACTATGCTTATTACGCTCAGGTTGCTGGTCTATACTATGGCATAAATCGCACAGATGGTGG
TTTCGAGGTGACACTCTTTGGTTATAATCACAAGTTAAGGATTCTGCTTGAAACTATAGTTGA
AAAGATTGCAGCATTTGAAGTGAAAACTGACAGGTTTTCAGTCATCAAGGAAATGGTAACC
AAGAAATATCAGAATATGAAATACCAACAACCTTATCAGCAGGCTATGTACTATTGCTCTTT
GATCTTACAAGATCATACCTGGCCTTGGACAGAACAACTCGACATACTTCCTGCTCTTCAAG
TTGAAGATATTGCTAAATTTGTACCATTAATGCTCTCGAGGACATTTTTAGACTTCTATATAG
CAGGGAACATTGAAAGCCATGAAGCAGAGTCGATAGTCAAGCACATTGATGATGCTCTCTTT
AATTGTTCAAAACCTGTGTGTAAGCCTTTATTCTCATCTCAACATTTGGCCAACCGAGTTGTT
AAACTTGAACGTGGCATGAGTTACTTCTACCCTTCAGAATGTCTTAATCCTGAAAATGAGAA
TTCTGCTCTTCTGCACTACATACAGGTTGGTCGTGATGACTTTAAGCTTAATGTTAAACTTCA
GCTTTTTGCTCTTGTTGCAAAGCAACCAACCTTTCATCAGCTACGATCTGTTGAGCAGCTGGG
GTACATTACTGTGCTCTTGCAGAGGAATGATTGTGGTATACGTGGACTACAGTTTATTATCCA
ATCCACAGTAAAGTCTCCCGGGAACATTGAACAAAGGGTTGAGGCATTTCTCAAAATGTTTG
AGACCAAGCTTTATGAGATGACAATTGATGAATTCAAG

Annotation in Phytozome:

Transcript Name: Phvul.001G133200.1

Description: METALLOPROTEASE

9. **CRG**: Glyceraldehyde-3-phosphate dehydrogenase C-subunit (GAPC)

Database: RefSeq (NCBI)

Sequence

>XM_003526927.3 PREDICTED: *Glycine max* glyceraldehyde-3-phosphate dehydrogenase, cytosolic (LOC100783902), mRNA

AGAAATCGCAAAAAAGGGTATAGCGTAATCCATGGCACAGTATCACAGGTCATACGCCGGT
CATGTAGGTGTCACCCACCCGACCCACCTACTTCACTTACCTTTACGTGCCATTTTGCTCTTTT
TTACCCTTCTTTCAACCCTTTATTAAAACCCTATCTCGCTCGCACTCACCCACACACCATCCG
TTCTAGTCTCAACTTCTGCTCTTCCAACTCCTACAACGCTTTTCTCTGCACTCATGGGCAAGG
TCAAGATCGGAATCAACGGATTTGGAAGAATTGGCCGTTTGGTTGCCAGAGTCGCTCTGCAA
AGAGACGATGTTGAACTCGTTGCCGTTAACGACCCTTTCATTACCACCGATTACATGACATA
CATGTTTAAATACGACAGTGTTCACGGACACTGGAAGCATCACGATGTCACCGTTAAGGACG
AGAAGACCCTTCTCTTCGGTGACAAGGCAGTCACTGTTTTTGGACACAGAAACCCTGAAGAG
ATCCCATGGGGGTCAACTGGAGCTGACATCATTGTTGAGTCCACCGGAGTTTTCACCGATAA
GGACAAGGCTGCCGCACATTTGAAGGGTGGTGCCAAGAAGGTTATTATTTCTGCCCCCAGTA
AGGATGCCCCCATGTTTGTTGTTGGTGTTAATGAGCACGAGTACAAGCCAGAGCTTGATATT
ATTTCCAACGCTAGCTGCACAACCAACTGCCTTGCTCCACTCGCCAAGGTTATCAATGACAG
GTTTGGCATTGTTGAGGGTTTGATGACCACTGTTCATTCCATCACTGCTACCCAGAAGACTGT
TGATGGGCCATCAGCCAAGGACTGGAGAGGTGGAAGAGCTGCTTCATTTAACATCATTCCTA
GCAGCACTGGAGCTGCCAAGGCTGTTGGGAAAGTCCTTCCTGCTTTGAATGGAAAATTGACT
GGTATGGCATTCCGTGTTCCCACCGTGGATGTCTCTGTTGTTGACCTCACGGTGAGGCTGGA
GAAGGAAGCCTCATATGATGAAATTAAAAATGCTATCAAGGAGGAATCAGAGGGCAAGTTG
AAGGGAATTCTTGGTTACACTGAAGATGATGTGGTCTCCACCGACTTTGTTGGTGATAACAG
ATCAAGTATTTTTGATGCAAAGGCTGGAATTGCATTGAATAAGAATTTTGTGAAGCTTGTCT
CTTGGTATGACAACGAGTGGGGATACAGTTCACGTGTCATTGACCTGCTTGTAATCGTTGCC
AAGAAGTCTTTTTAATTAAGGTGGTTCTAACATTATGTGCTGGTCAAGCTGCGATTTAGTGTC
TTGCTCGTGCAAAAATTGAGAAGTATGAATAAATCGGTTTCCGGAACCAGTGTTGTTACCCT
GTTACGAGCATTAAGCTCTTTTTTGTTGCCTCTTCTGTGTAGGGGATGGAGTTTTTTGGATTCT
TATATACTTGCAGATGTACTCGGGCTTTATTGATTCTGATAGCAGACATTTTCCCTCCATTTTT
CATCCATGGTTCTCGAGTGTTTGAACCCCCCTTCCTTTTCTGCTTTGAATTAAAGATTCGTGTT
TTATATA

Annotation in Phytozome:

Transcript Name: Glyma.06G172600.1

Description: PTHR10836//PTHR10836:SF44 - GLYCERALDEHYDE 3-PHOSPHATE DEHYDROGENASE
