# Supplementary material for: Cowpea and abiotic stresses: identification of reference genes for transcriptional profiling by qPCR
Source: Plant Methods. 2018 Oct 12;14:88. doi: 10.1186/s13007-018-0354-z (PMC6182843; doi:10.1186/s13007-018-0354-z)
Supplement: Supplementary file 4 — Additional file 4. Table S1. MIQE checklist for reviewers, and editors. All essential information (E) must be submitted with the manuscript. Desirable information (D) should be submitted if available. [file 13007_2018_354_MOESM4_ESM.docx]

**Table S1. MIQE checklist for authors, reviewers and editors. All essential information (E) must be submitted with the manuscript. Desirable information (D) should be submitted if available.**

| **ITEM TO CHECK** | **Importance** | **Checklist** | **Yes/No/Not Applicable (NA)** | **DESCRIPTION** |
| --- | --- | --- | --- | --- |
| **EXPERIMENTAL DESIGN** |  |  |  |  |
| Definition of experimental and control groups | **E** | **ok** | **Yes** | Two trials were carried out in the present work. For root dehydration, the control was maintained under constant hydration; the treatments consisted of dehydration of the roots exposed to air during the stress times employed (25, 50, 75, 100, 125, 150 min). For the qPCR, the treatments of 25, 75 and 150 min were analyzed. Concerning the salt stress trial, the control was maintained under irrigation with 200 mL of Hoagland's Solution; for the treatments, NaCl was added to the Hoagland's Solution to a final concentration of 150 mM. Roots were collected at 30, 60 and 90 minutes after contact with such solution. The referred times were analyzed via qPCR. |
| Number within each group | **E** | **ok** | **Yes** | Three replicates were used for each treatment; each biological replicate was composed of five different plants. |
| Assay carried out by core lab or investigator's lab? | **D** | **ok** | **Yes** | Investigator's lab. |
| Acknowledgement of authors' contributions | **D** | **ok** | **Yes** | Conceived and designed the experiments: AMBI, EAK, and MSG; Performed the experiments and analyzed the data: LLBA, JRCFN, VP, MKSM, FTA, MGS, EAK and JPBN; Contributed reagents / materials / analysis tools: AMBI, EAK; Wrote the manuscript: LLBA, JCRFN, and AMBI. AMBI coordinated the research project. |
| **SAMPLE** |  |  |  |  |
| Description | **E** | **ok** | **Yes** | Each biological sample was from cowpea roots. Seeds were pre-germinated on moist filter paper, in the dark at 25°C ± 1°C, and in 65% ± 5% relative humidity. After 15 days, seedlings with the first trifoliate leaf fully developed (V2 developmental stage) were submitted to analyzed stresses (see Material and Methods section). |
| Volume/mass of sample processed | **D** | − | **NA** | − |
| Microdissection or macrodissection | **E** | − | **NA** | − |
| Processing procedure | **E** | **ok** | **Yes** | Upon collection of root tissue, each sample was packed in aluminum foil and then dipped in liquid nitrogen. Next, the samples were immediately stored at -80 ^o^C in a deep freezer. |
| If frozen - how and how quickly? | **E** | **ok** | **Yes** | The root tissue was collected from the control and from each treatment performed, being immediately dipped in liquid nitrogen. |
| If fixed - with what, how quickly? | **E** | − | **NA** | − |
| Sample storage conditions and duration (especially for FFPE samples) | **E** | **ok** | **Yes** | Samples were stored at -80oC in deep freezer for two weeks before being immediately processed for RNA extraction upon removal from the deep freezer. |
| **NUCLEIC ACID EXTRACTION** |  |  |  |  |
| Procedure and/or instrumentation | **E** | **ok** | **Yes** | SV Total RNA Isolation System. |
| Name of kit and details of any modifications | **E** | **ok** | **Yes** | Cat.#Z3105 and protocol supplied by manufacturer. |
| Source of additional reagents used | **D** | − | **NA** | − |
| Details of DNase or RNAse treatment | **E** | **ok** | **Yes** | See SV Total RNA Isolation System protocol. |
| Contamination assessment (DNA or RNA) | **E** | **ok** | **Yes** | Using the ratio of 260 nm/280 nm. |
| Nucleic acid quantification | **E** | **ok** | **Yes** | Nanodrop©2000 (Thermo Scientific). |
| Instrument and method | **E** | **ok** | **Yes** | Spectrophotometric analysis with measurements at 280 nm and 260 nm. Agarose gel electrophoresis at 2% agarose for each sample. |
| Purity (A260/A280) | **D** | **ok** | **Yes** | 1.8 < Purity < 2.0 |
| Yield | **D** | **ok** | **Yes** | Variable, depending on the treatment investigated. Information available upon request. |
| RNA integrity method/instrument | **E** | **ok** | **Yes** | Gel electrophoresis at 2% agarose and Nanodrop©2000 (Thermo Scientific). |
| RIN/RQI or Cq of 3' and 5' transcripts | **E** | − | **No** | − |
| Electrophoresis traces | **D** | **ok** | **Yes** | Pictures are available upon request. |
| Inhibition testing (Cq dilutions, spike or other) | **E** | **ok** | **Yes** | Using the formula E% = [−1 + 10^(−1/slope)^] × 100 and testing dilution series of sample cDNA, incorporating several orders of magnitude (100 – 0.1 ng) and C_q_ values were plotted against log_10_[cDNA quantity]. |
| **REVERSE TRANSCRIPTION** |  |  |  |  |
| Complete reaction conditions | **E** | **ok** | **Yes** | For each sample, 1 µg of the RNA was reversed-transcribed into cDNA using the Improm-IITM Reverse Transcriptional System (Promega) with oligo(dT) primers following the manufacture’s recommendations. |
| Amount of RNA and reaction volume | **E** | **ok** | **Yes** | 1 μg of total RNA per 20 μL reaction volume for each reverse transcription reaction. |
| Priming oligonucleotide (if using GSP) and concentration | **E** | − | **NA** | − |
| Reverse transcriptase and concentration | **E** | **ok** | **Yes** | The ImProm-II™ Reverse Transcriptase (Cat.# A3803) supplied with this system is formulated for efficient first-strand cDNA synthesis or RT-PCR applications. For robust activity was used 1µL of the enzyme in a 20 µL reaction. |
| Temperature and time | **E** | **ok** | **Yes** | 42 ^o^C for 60 min. |
| Manufacturer of reagents and catalogue numbers | **D** | **ok** | **Yes** | See ImProm-II™ Reverse Transcription System protocol (link: https://www.promega.com/~/media/files/resources/protocols/technical%20manuals/0/improm-ii%20reverse%20transcription%20system%20protocol.pdf) |
| Cqs with and without RT | **D** | **ok** | **Yes** | See Supplementary Tables 2 and 3. |
| Storage conditions of cDNA | **D** | **ok** | **Yes** | Permanently at -20 °C in sterile test tubes. |
| **qPCR TARGET INFORMATION** |  |  |  |  |
| If multiplex, efficiency and LOD of each assay. | **E** | − | **NA** | − |
| Sequence accession number | **E** | **ok** | **Yes** | See Supplementary Appendix 1 and Table 1 for the sequence accession numbers of the CRGs and target genes. |
| Location of amplicon | **D** | **ok** | **Yes** | Preferably distinct exons (Table 1). |
| Amplicon length | **E** | **ok** | **Yes** | 117 to 192 nt. |
| *In silico* specificity screen (BLAST, etc.) | **E** | **ok** | **Yes** | Used primer-BLAST against Angiosperms as target organism (data not show in the manuscript). |
| Pseudogenes, retropseudogenes or other homologs? | **D** | **ok** | **Yes** | Pseudogenes were excluded from the analysis. |
| Sequence alignment | **D** | **ok** | **Yes** | Alignment available upon request. |
| Secondary structure analysis of amplicon | **D** | − | **No** | - |
| Location of each primer by exon or intron (if applicable) | **E** | **ok** | **Yes** | Care was taken to have the primer sequence spanning exon boundaries (Table 1). However, not all primers presented such an anchoring pattern. Aiming to reinforce the analysis of quality and purity of the analyzed RNA population, a pair of Actin primers (that anchors within an intron) was used. The functionality of this primer pair would indicate the presence of genomic DNA in the reaction. |
| What splice variants are targeted? | **E** | **ok** | **Yes** | No splice variants were targeted because there is no information about them in the analyzed organism. |
| **qPCR OLIGONUCLEOTIDES** |  |  |  |  |
| Primer sequences | **E** | **ok** | **Yes** | See Table 1 for the sequences of the 13 primer pairs used. |
| RTPrimerDB Identification Number | **D** | − | **NA** | - |
| Probe sequences | **D** | − | **NA** | - |
| Location and identity of any modifications | **E** | − | **NA** | No modifications. |
| Manufacturer of oligonucleotides | **D** | **ok** | **Yes** | Sinapse Biotecnologia. |
| Purification method | **D** | **ok** | **Yes** | Desalted. |
| **qPCR PROTOCOL** |  |  |  |  |
| Complete reaction conditions | **E** | **ok** | **Yes** | The qPCR amplifications were performed on the LineGene 9660 model (Bioer), using SYBR Green detection. Each reaction mixture comprised 1 μL of template cDNA (diluted 5-fold), 5 μL of HotStart-IT SYBR Green qPCR Master Mix 2x (USB), 0.05 μL of ROX, 1.95 μL of water, and 1 μL primer (500 nM each) to a final volume of 10 μL. The reactions were denatured at 95°C for 2 min, followed by 40 cycles of 95°C for 15 s, 58°C for 30 s, and 72°C for 15 s in 96-well reaction plates, with the detection of the fluorescence signal at the end of each extension step. After amplification, dissociation curves were produced (60 °C to 95 °C at a heating rate of 0.1 °C/sec and acquiring fluorescence data every 0.3 °C) to discriminate the main reaction products from other nonspecific ones or primer-dimers. |
| Reaction volume and amount of cDNA/DNA | **E** | **ok** | **Yes** | 20 ng of cDNA in 10 μL reaction volume. |
| Primer, (probe), Mg++ and dNTP concentrations | **E** | **ok** | **Yes** | Final concentration of 500 nM for each primer in the reaction, 2.5 mM MgCl_2_ and dNTP concentration provided with the Master mix (proprietary information). |
| Polymerase identity and concentration | **E** | **ok** | **Yes** | HotStart-IT Taq DNA Polymerase [HotStart-IT SYBR Green qPCR Master Mix 2x (USB)]. |
| Buffer/kit identity and manufacturer | **E** | **ok** | **Yes** | HotStart-IT SYBR Green qPCR Master Mix 2x (USB). |
| Exact chemical constitution of the buffer | **D** | **ok** | **Yes** | The mix combines USB HotStart-IT Taq DNA Polymerase, heatlabile UDG, SYBR Green I, MgCl2, and Ultrapure nucleotides with an optimized dUTP to dTTP ratio in a unique buffer formulation. Magnesium and nucleotide concentrations are 5 mM and 0.4 mM each, respectively [HotStart-IT SYBR Green qPCR Master Mix 2x (USB)]. |
| Additives (SYBR Green I, DMSO, etc.) | **E** | **ok** | **Yes** | SYBR Green I, ROX Passive Reference Dye, and Fluorescein Passive Reference Dye. |
| Manufacturer of plates/tubes and catalog number | **D** | **ok** | **Yes** | MicroAmp® Fast Optical 96-Well Reaction Plate with Barcode, 0.1 mL, Cat No.: 4346906. |
| Complete thermocycling parameters | **E** | **ok** | **Yes** | 95 °C for 2 min as an initial step followed by 40 cycles of 95 °C for 15 s, 58 °C for 60 s and 72º for 15 s . After amplification, dissociation curves were produced (60 °C to 95 °C at a heating rate of 0.1 °C/sec and acquiring fluorescence data every 0.3 °C) to discriminate the main reaction products from other nonspecific ones or primer-dimers. |
| Reaction setup (manual/robotic) | **D** | **ok** | **Yes** | Manual |
| Manufacturer of qPCR instrument | **E** | **ok** | **Yes** | LineGene 9660 model (Bioer). |
| **qPCR VALIDATION** |  |  |  |  |
| Evidence of optimisation (from gradients) | **D** | − | **No** | No optimization steps were realized. |
| Specificity (gel, sequence, melt, or digest) | **E** | **ok** | **Yes** | Melting curves for each biological replicate (see Supplementary Appendix 2). |
| For SYBR Green I, Cq of the NTC | **E** | **ok** | **Yes** | See Additional files 6 and 7 |
| Standard curves with slope and y-intercept | **E** | **ok** | **Yes** | See Table 4. |
| PCR efficiency calculated from slope | **E** | **ok** | **Yes** | See Table 4. |
| Confidence interval for PCR efficiency or standard error | **D** | − | − | - |
| r2 of standard curve | **E** | **ok** | **Yes** | > 0.982. |
| Linear dynamic range | **E** | **ok** | **Yes** | 13.5 to 25.9 Cycles. |
| Cq variation at lower limit | **E** | **ok** | **Yes** | %CV=2.18. |
| Confidence intervals throughout range | **D** | − | − | - |
| Evidence for limit of detection | **E** | − | − | - |
| If multiplex, efficiency and LOD of each assay. | **E** | − | **NA** | - |
| **DATA ANALYSIS** |  |  |  |  |
| qPCR analysis program (source, version) | **E** | **ok** | **Yes** | LineGene 9660 Software (Bioer). |
| Cq method determination | **E** | **ok** | **Yes** | Manual, set for all assays at ΔRn=0.25. |
| Outlier identification and disposition | **E** | **ok** | **Yes** | Through standard curve: Cq= -3.225*(log10template copies) +35.25) and subsequent analysis with GraphPad Prism V.6 for Macintosh. |
| Results of NTCs | **E** | **ok** | **Yes** | > 37 or no amplification. |
| Justification of number and choice of reference genes | **E** | **ok** | **Yes** | Although statistical data from the geNorm software indicate the use of two reference genes as suitable to obtain a normalization factor, three reference genes were used. Such quantitative was employment following the instructions of Vandesompele et al. (2002). |
| Description of normalisation method | **E** | **ok** | **Yes** | Four strategies (geNorm, NormFinder, BesKeeper, and Delta-Ct method) were used to indicate the reference genes used in the present study. |
| Number and concordance of biological replicates | **D** | **ok** | **Yes** | There were 3 biological replicates of each analyzed treatment. |
| Number and stage (RT or qPCR) of technical replicates | **E** | **ok** | **Yes** | There were 3 technical replicates for each biological replicate. |
| Repeatability (intra-assay variation) | **E** | **ok** | **Yes** | Dependent of the analyzed transcript. |
| Reproducibility (inter-assay variation, %CV) | **D** | **ok** | **Yes** | 95% Confidence Intervals, Intercept=33.12 to 38.03, Slope= -3.18 to -3.43. |
| Power analysis | **D** | − | **No** | - |
| Statistical methods for result significance | **E** | **ok** | **Yes** | For the reference gene indication, the statistical methods were contained in the geNorm, NormFinder, BestKeeper algorithms and Delta-Ct method; For relative expression analysis: REST2009 software (p<0.05). |
| Software (source, version) | **E** | **ok** | **Yes** | See Material and Methods. |
| Cq or raw data submission using RDML | **D** | **ok** | **Yes** | See Additional files 6 and 7. |

Legend for abbreviations: NA= not applicable.
