# Supplementary material for: Cowpea and abiotic stresses: identification of reference genes for transcriptional profiling by qPCR
Source: Plant Methods. 2018 Oct 12;14:88. doi: 10.1186/s13007-018-0354-z (PMC6182843; doi:10.1186/s13007-018-0354-z)
Supplement: Supplementary file 6 — Additional file 6. Table S2. Cqs obtained for the candidate reference genes analyzed for the root dehydration assay. [file 13007_2018_354_MOESM6_ESM.docx]

| **Table S2. Cqs obtained for the candidate reference genes analyzed for the root dehydration assay in cowpea** (*Vigna unguiculata*). | | | | | | | |
| --- | --- | --- | --- | --- | --- | --- | --- |
| **Cultivar / Treatment** | CRG 1 | CRG 2 | CRG 3 | CRG 4 | CRG 5 | CRG 6 | CRG 7 |
|  | ***β-TUB*** | ***EF1-α*** | ***VuACT*** | ***UE21D*** | ***UNK*** | ***FBOX*** | ***VuUBQ10*** |
| Santo Inácio Control | 19.40 | 15.80 | 21.40 | 18.40 | 19.70 | 21.60 | 16.30 |
| Santo Inácio Control | 19.90 | 17.00 | 21.40 | 19.00 | 20.30 | 22.50 | 17.40 |
| Santo Inácio Control | 18.60 | 16.37 | 21.00 | 18.20 | 19.30 | 21.30 | 15.80 |
| Pingo de Ouro Control | 19.70 | 16.70 | 21.70 | 18.90 | 19.60 | 21.90 | 16.80 |
| Pingo de Ouro Control | 19.40 | 16.50 | 21.20 | 18.60 | 19.50 | 21.70 | 16.30 |
| Pingo de Ouro Control | 20.20 | 17.10 | 21.80 | 18.70 | 20.40 | 22.10 | 17.00 |
| Santo Inácio Treatment 25 min | 19.60 | 16.30 | 21.50 | 18.80 | 19.90 | 21.30 | 16.70 |
| Santo Inácio Treatment 25 min | 20.20 | 16.70 | 22.00 | 19.40 | 20.40 | 21.80 | 16.40 |
| Santo Inácio Treatment 25 min | 19.00 | 16.37 | 21.20 | 18.50 | 19.80 | 21.80 | 16.00 |
| Pingo de Ouro Treatment 25 min | 20.60 | 17.00 | 21.90 | 19.60 | 20.70 | 22.60 | 16.90 |
| Pingo de Ouro Treatment 25 min | 19.00 | 16.20 | 20.70 | 18.10 | 19.60 | 21.50 | 16.00 |
| Pingo de Ouro Treatment 25 min | 20.00 | 18.50 | 20.60 | 18.07 | 19.50 | 21.20 | 16.40 |
| Santo Inácio Treatment 75 min | 20.50 | 16.80 | 22.50 | 19.30 | 20.90 | 22.80 | 16.90 |
| Santo Inácio Treatment 75 min | 19.40 | 16.20 | 21.80 | 19.00 | 19.90 | 22.10 | 16.40 |
| Santo Inácio Treatment 75 min | 19.70 | 16.60 | 21.70 | 19.90 | 20.40 | 22.10 | 16.00 |
| Pingo de Ouro Treatment 75 min | 21.20 | 18.30 | 23.30 | 20.10 | 21.50 | 23.70 | 17.30 |
| Pingo de Ouro Treatment 75 min | 20.50 | 17.20 | 22.20 | 19.40 | 21.10 | 22.90 | 17.10 |
| Pingo de Ouro Treatment 75 min | 19.80 | 17.50 | 20.80 | 18.40 | 19.90 | 21.90 | 15.90 |
| Santo Inácio Treatment 150 min | 21.30 | 16.53 | 23.50 | 20.40 | 22.20 | 22.90 | 17.90 |
| Santo Inácio Treatment 150 min | 20.80 | 17.00 | 22.70 | 19.80 | 21.40 | 23.20 | 16.90 |
| Santo Inácio Treatment 150 min | 19.40 | 16.50 | 21.50 | 19.10 | 20.80 | 21.60 | 16.10 |
| Pingo de Ouro Treatment 150 min | 19.80 | 17.00 | 21.70 | 18.90 | 20.20 | 22.50 | 16.10 |
| Pingo de Ouro Treatment 150 min | 20.60 | 17.50 | 22.00 | 19.20 | 20.80 | 21.90 | 16.80 |
| Pingo de Ouro Treatment 150 min | 20.10 | 16.70 | 22.00 | 19.40 | 21.00 | 23.10 | 16.40 |
| Mean | 19.95 | 16.85 | 21.75 | 19.05 | 20.37 | 22.17 | 16.58 |
| **NTC** | 39.30 | 38.50 | 39.20 | − | − | 37.80 | − |
| Legend: CRG (Candidate Reference Gene); NTC (no template control); *Vu* (*Vigna unguiculata*); *β-TUB* (beta-tubulin); *EF1-α* (elongation factor 1-alfa); VuACT (actin); *UE21D* (ubiquitin-conjugating enzyme E2 variant 1D); *UNK* (unknown); *FBOX* (F-box protein); *VuUbq10* (polyubiquitin 10). | | | | | | | |
