# Supplementary material for: Cowpea and abiotic stresses: identification of reference genes for transcriptional profiling by qPCR
Source: Plant Methods. 2018 Oct 12;14:88. doi: 10.1186/s13007-018-0354-z (PMC6182843; doi:10.1186/s13007-018-0354-z)
Supplement: Supplementary file 8 — Additional file 8. Table S4. Data explored by REST software to analyze the relative expression of target transcripts in cowpea under root dehydration stress. [file 13007_2018_354_MOESM8_ESM.docx]

**Legend:** TRG (target gene); Std. Error (standard error); 95% C.I. (95% Confidence Intervals); P(H1) (Hypothesis Test); UR (up-regulated at p < 0.05); DR (down-regulated at p < 0.05); ns (not significant at the level of p ≤ 0.05); *Vu* (*Vigna unguiculata*); *VuCHiB* (chitinase B); *VuLTP* (lipid transfer protein); *VuCHI* (chalcone isomerase); *VuCHS* (chalcone synthase). Santo Inácio (sensitive accession); Pingo de Ouro (tolerant accession).
